# Massive losses and gains of northern land carbon stocks since the Last Glacial Maximum

**DOI:** 10.1126/sciadv.adt6231

**Published:** 2025-08-29

**Authors:** Amelie Lindgren, Peter Kuhry, Max Holloway, Zhengyao Lu, George Tanski, Gustaf Hugelius

**Affiliations:** ^1^ Department of Physical Geography, Stockholm University, Stockholm, Sweden.; ^2^ Department Earth Science, University of Gothenburg, Gothenburg, Sweden.; ^3^ Bolin Centre for Climate Research, Stockholm University, Stockholm, Sweden.; ^4^ Planetary Technologies, 24 Orion Ct, Dartmouth, NS B2Y 4W6, Canada.; ^5^ Department of Physical Geography and Ecosystem Science, Lund University, Lund, Sweden.; ^6^ Department of Earth Sciences, Vrije Universiteit Amsterdam, Amsterdam, Netherlands.; ^7^ Permafrost Research Unit, Alfred Wegener Institute Helmholtz Centre for Polar and Marine Research, Telegrafenberg, 14473 Potsdam, Germany.

## Abstract

The dynamics of atmospheric CO_2_ concentrations during and following the last deglaciation have mainly been ascribed to carbon release from and uptake in oceans, primarily in the Southern Ocean. But recent studies also point toward a terrestrial influence. We quantify dynamic changes to northern terrestrial carbon stocks from the Last Glacial Maximum (21,000 years) until present at millennial time steps using a combination of paleo-data and climate-biome modeling. During the deglaciation, northern land carbon storage declined by >300 petagrams of carbon with a minimum around 11,000 years, followed by progressively higher land carbon stocks during the Holocene. We find evidence that dynamic changes in terrestrial land carbon stocks were of a scale to exert large influence on atmospheric CO_2_ concentrations and that postglacial terrestrial carbon stock dynamics were dominated by losses from permafrost-affected loess and gains into peatlands.

## Teaser

Carbon losses from permafrost influenced the atmospheric CO_2_ when temperatures increased after the last ice age.

## INTRODUCTION

Ice core records show that atmospheric CO_2_ has decreased during glacial periods and increased during interglacial periods for the past 800,000 years (*1*), revealing that past warming has coincided with elevated CO_2_ (*2*). Understanding the drivers behind this natural cycle, with changes of about 100 parts per million (ppm) between cold and warm periods, is important to accurately project the climate system response to ongoing and future global warming. The most commonly invoked hypothesis is that the Southern Ocean played a dominant role in regulating the atmospheric CO_2_ variations (*3*, *4*). It has been argued that the ocean carried the burden of not only storing the excess atmospheric CO_2_ at the cold Last Glacial Maximum (LGM) but also stored ~300 to ~450 Pg carbon (C) from the terrestrial biosphere (*5*–*7*). This implies a substantial net flux of C from the ocean to the atmosphere, and to the terrestrial biosphere, between the LGM and present day. This hypothesis is plausible and supported by evidence (*8*). However, centennial-scale records have shown rapid jumps in atmospheric CO_2_ and methane (CH_4_) during the deglaciation (*9*), which, together with isotopic constraints, point toward emissions of C from land (*10*). It is possible that land acted as a C source to the atmosphere during pivotal phases of the deglaciation, although the overall land storage shows an increase between the LGM and present day (*11*–*16*). It is known that permafrost degradation (*17*) and coastal erosion (*18*) can cause abrupt remobilization and release of C, which could be invoked to partially explain periods of rapid atmospheric CO_2_ increase at around 16.3 ka (1 ka = 1000 calibrated years before the present), 14.8 and 11.7 ka (*9*). Evidence is mounting that near-shore or onshore permafrost processes caused the remobilization of large amounts of soil carbon to the Arctic Ocean sediments around these time periods (*19*, *20*), suggesting that the terrestrial soil C storage not increased linearly from the LGM to present but changed dynamically and also underwent losses.

In this study, we reconstruct millennial-scale terrestrial soil C storage and change in the extratropical north (>23° latitude) from the LGM [21 thousand years (ka)] until preindustrial (PI, hereafter also denoted as 0 ka). We do not account for human activities, changes in fire regimes or lakes, although these may have been important (*21*, *22*). We also disregard changes in phytomass C as this pool is considered to be comparatively small (*13*). We capture broad-scale climate-driven transitions that we propose played an important role in the postglacial terrestrial soil C dynamics: shifts of biome zones, permafrost thaw of both near surface soils (0- to 2-m depth), and deep deposits, melting ice sheets, inundation of continental shelves by rising seas, and peatland expansion and development (*23*). To the extent possible, these millennial-scale reconstructions are based on observational paleo-data combined with a mix of machine learning and dynamic modeling.

## RESULTS

### Soil carbon stocks

**Fig. 1.**
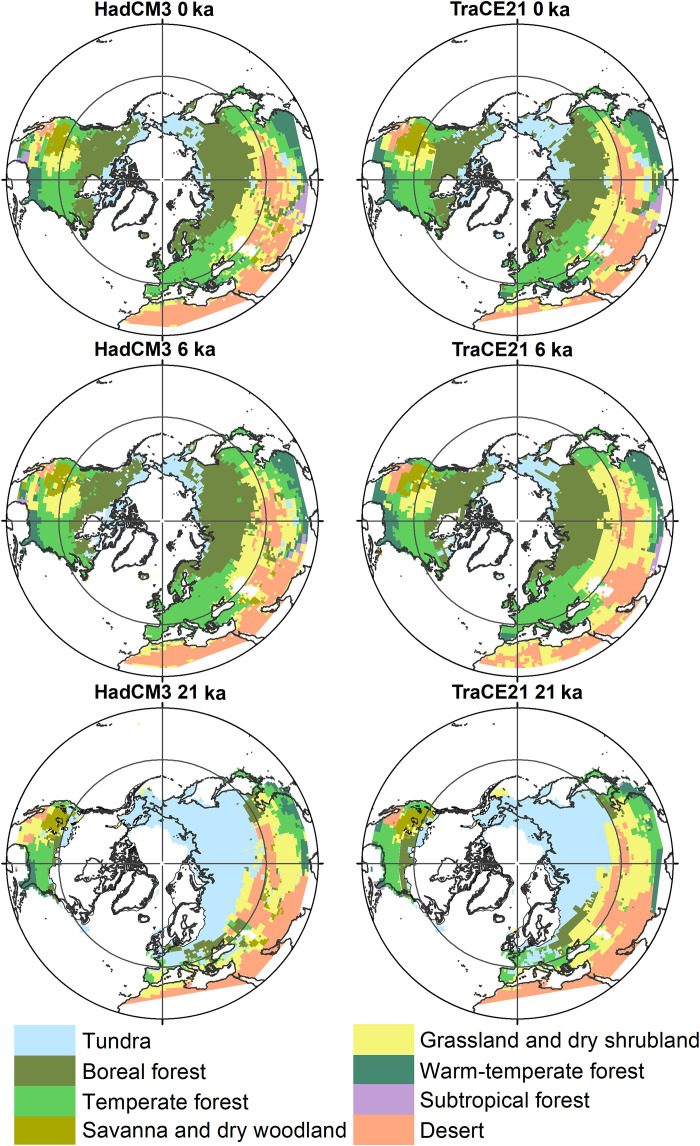
Reconstructed biome distributions at 0, 6, and 21 ka. Biomes were modeled with simulated climate from HadCM3 and CCSM3 TraCE-21k. The ice sheets of the LGM at 21 ka are included in the bottom. Similar data were created for each millennium from 21 to 0 ka.

**Fig. 2.**
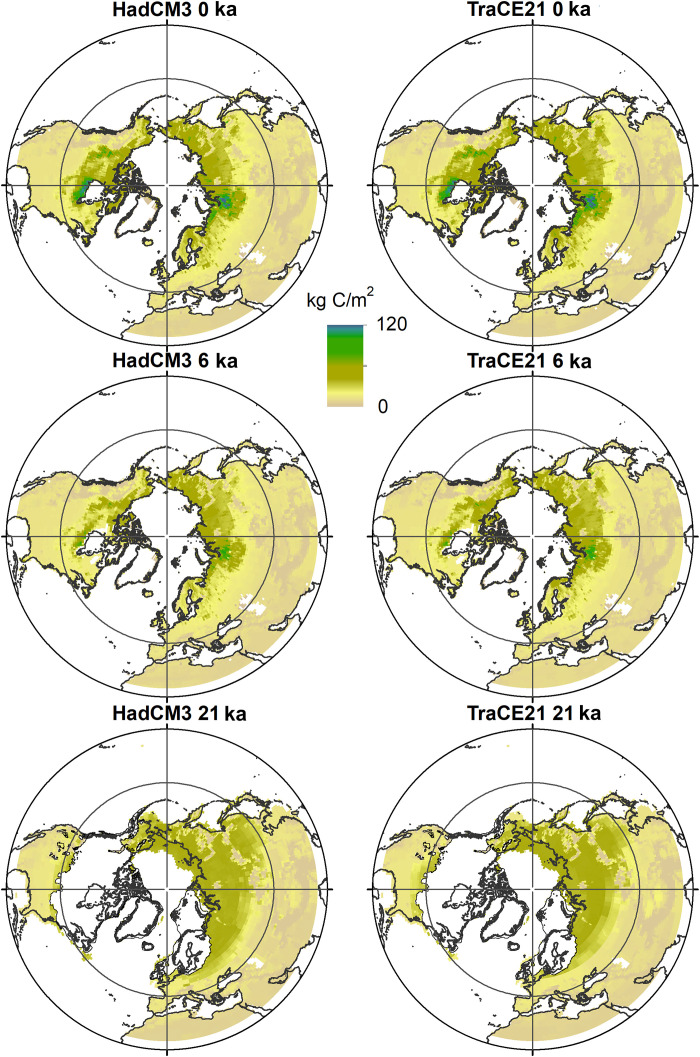
Distribution of soil C density. Storage of mineral and peat soil C in Pg C for each grid cell (1° resolution) at 0, 6, and 21 ka. Results are based on probability distributions of biomes and their typical C content in kilograms of carbon per square meter. Biomes were modeled with climate from HadCM3 and CCSM3 TraCE-21k. The ice sheets of the LGM (21 ka) are included in the bottom. Deep loess C is not included.

**Fig. 3.**
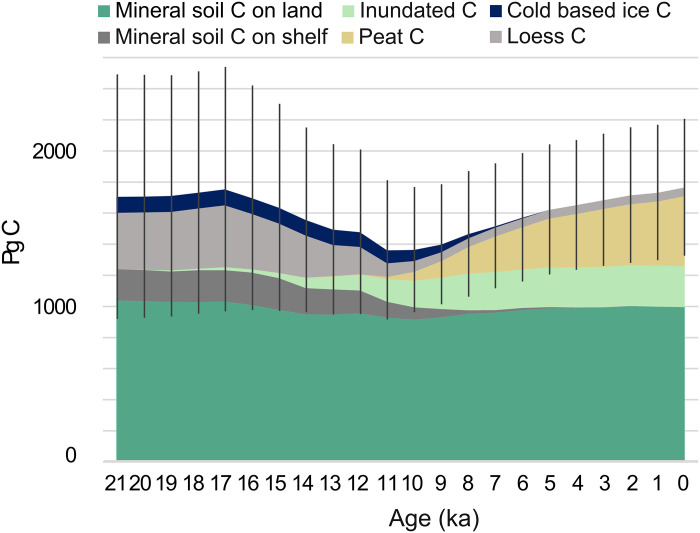
Time series of the development of land C storage. Land C storage in Pg C, north of the 23rd parallel, for each millennium between the LGM (21 ka) and PI (here denoted as 0 ka), calculated as the average between biome reconstructions based on HadCM3 and TraCE climate simulations. Stocks are divided into categories of soil C on land (soil C = 0 to 2 m storage in mineral soils), soil C on exposed sea shelves, soil C on inundated shelves, peat soil C, loess deposit C below 2 m depth, and soil C released from beneath cold based ice as the ice sheets retreat. Uncertainty estimates are propagated from SDs of each stock.

Over broad spatial scales, soil C stocks can be predicted from vegetation zones, or biomes (table S1), as these zones reflect differences in environmental conditions and productivity (*11*, *13*, *15*). For this study, we used geographical databases of modern mineral soil C stocks (*24*, *25*) and averaged these for each biome (*26*). As the climate changed during the deglaciation, biomes shifted in their extent and geographical location (Fig. 1). Vast territories of C-rich tundra underlain by permafrost were replaced by forested ecosystems with lesser soil C storage. Our reconstructions of past mineral soil C stocks in different biome types relies on the use of modern ecosystems as analogs for past ecosystem assemblages. With geographical shifts of biomes, we reconstruct a substantial redistribution of soil C across the domain (Fig. 2), as well as a decrease of C storage per square meter in regions where permafrost degraded (fig. S1). However, for most millennia, this redistribution of C exceeded the overall net losses to the atmosphere with several hundred percent (table S2), and our millennial-scale biome and permafrost reconstructions indicate modest changes in mineral soil C stocks in relation to the total stock size (Fig. 3 and table S3). Deglacial (21 to 12 ka) and Holocene (12 to 0 ka) land C storage changes were instead largely driven by the loss of C from deep loess deposits and the sequestration of C into peatlands.

### Loess deposits

Under permafrost conditions, C can accumulate into deposits of accumulated dust particles, also known as loess (*27*). Once permafrost retreats from these areas, much of this C is lost. This suggested change is derived from the differences between modern permafrost-affected ice-rich loess deposits (Yedoma) and loess deposits outside the current permafrost extent. The loess deposits relevant for this study are located in North America, Central Europe, Central Siberia, and China [map available in (*13*)]. We find that most C losses from thawing permafrost loess occurred before 10 ka and that the overall change accounts for the largest net C loss during the deglaciation (~340 Pg C; Fig. 3 and table S4).

A previous estimate for C storage in deep loess deposits outside the current Yedoma region, but located within the limits of the northern permafrost region during the LGM, was 366 Pg C of which only 48 Pg C remains after permafrost thaw in the loess deposits today (*13*). This number was based on an additional loess area of 2.66 million km^2^ for a depth of >3 m, to avoid double accounting with biome stocks between 0 and 3 m (*13*).

In the current study, we calculate a mean C storage in deep loess deposits at 21 ka of 363 (324 to 402 SD) Pg C. On the one hand, the additional mean loess area of 2.30 (2.07 to 2.52) million km^2^ is somewhat lower than in the original analysis (*13*); on the other hand, an extra 1 m of original deposit was added because biome C stocks have only been calculated down to 2 m. Only 57 (44 to 70) Pg C of the estimated LGM stock remains in these loess deposits today.

### Peatlands

Peatlands, which are the most space efficient C stores on land, are thought to have been rare during glacial times (*28*). However, once the deglaciation progressed into the Holocene, peatlands expanded rapidly and began to accumulate C (*29*). The growth of peat into present times is estimated to 450 ± 162 Pg C (table S5) and accounts for the only net C sequestration on land from the atmosphere in our study. It should be noted that continuous drawdown of atmospheric CO_2_ into new and existing peatlands took place also, while concentrations of atmospheric CO_2_ remained stable (see Discussion), thereby allowing for CO_2_ release from other sources during the Holocene.

**Fig. 4.**
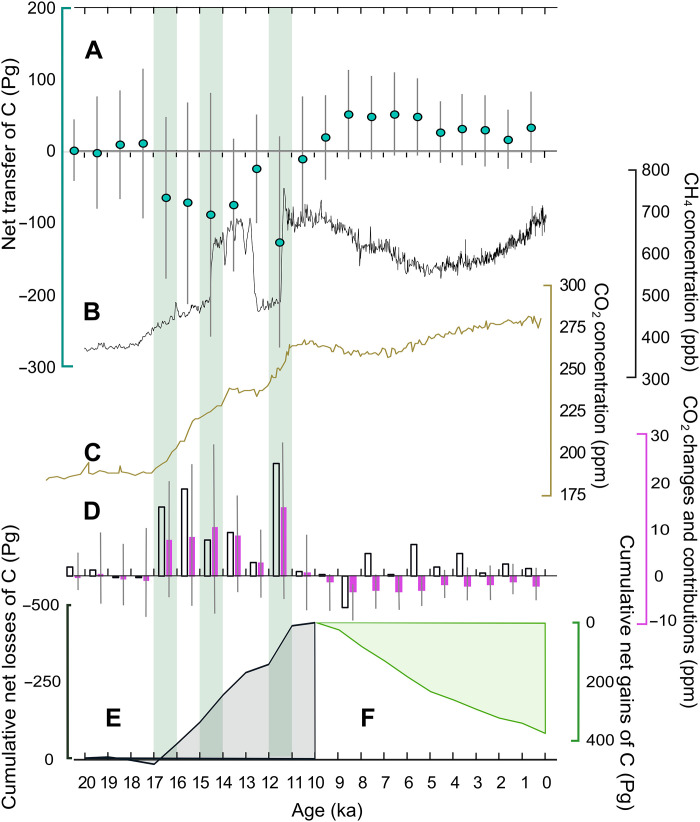
Time series of net land C transfers, atmospheric CO_2_ and CH_4_ concentrations, and cumulative losses and gains of C. (**A**) Blue circles show changes in C stock from the previous millennium with uncertainties estimated from additive error propagation. Negative numbers indicate a transfer of C from the land system to the atmosphere. (**B**) Black: Deglacial composite curve of atmospheric CH_4_ concentration (*9*, *118*). (**C**) Beige: Deglacial curve of atmospheric CO_2_ concentration from Dome Concordia, Antarctica (*117*). (**D**) Millennial-scale change in atmospheric CO_2_ concentration (white bars) (*117*), compared to estimated parts per million development from net transfers of land C (purple) with error bars. (**E**) Cumulative net gains and losses of C from land between 21 and 10 ka in Pg C. (**F**) Cumulative net gains to land from atmosphere between 9 and 0 ka in Pg C. Light green bars indicate millennia bracketing known periods of rapid carbon transfer from land. ppb, parts per billion.

### Effect of ice sheet retreat

The most marked change that occurred on land after the LGM was the loss of the continental ice sheets. The ice sheet retreat in both Eurasia and North America (*30*, *31*) had a direct impact on the soil C storage in three ways. First, the retreat resulted in losses from a subglacial soil C storage (*13*). Second, the retreat exposed new land for soil development with C accrual (*32*) and permafrost aggradation. Overall, these areas saw a net increase in C storage once the ice retreated, compensating for losses occurring elsewhere in the domain. Third, the melting ice sheets also caused sea-level rise and widespread inundation of soils on continental shelves (*13*, *33*).

The fate of the soil C on continental shelves as sea level rose remains elusive. Not only is the initial C storage uncertain, but it is also unknown whether coastal erosion caused widespread permafrost collapse, or whether gradual sea level rise preserved C stocks in place as part of the subsea permafrost system. Permafrost collapse occurs today in coastal regions (*17*), but preserved ice-bonded terrestrial permafrost in continental shelf oceans suggests that noncatastrophic inundation occurred (*34*).

We conservatively assume that all inundated C remains in our C stock estimate and that possible losses have been compensated by later deposition of sediments (*13*, *35*). We estimate the storage of C on the inundated shelf at PI to be higher than the storage on the fully exposed shelf at the LGM (table S6). This means that in our reconstruction, carbon storage on the exposed shelves increased during the deglacial period before inundation. This both disagrees and agrees with the literature on the topic, where losses from the LGM to present have been reported for the shelf permafrost C, but where the total stock on the inundated shelf increases to present due to sedimentation of C-rich material (*35*). Although the stock remains in our analysis, we account for the shift of C from a stock of soil C on exposed shelfs to an inundated stock (Fig. 3). The new land exposed by ice retreat was almost equal to the area of land that was simultaneously inundated by sea level rise within the domain. This means that the total area with mineral soil or sediment C storage increased from the LGM to PI within our domain, as we do not discount the inundated area. Consequently, the C storage per area decreased (fig. S1) as mineral C stocks have remained relatively constant.

### Stock changes

The limited growth of stored soil C between the LGM and PI found in this study (+60 Pg C) does not agree with the results in a previous similar study (*13*), which indicated a gain of about 400 Pg C. An analysis of the discrepancies between the previous findings (*13*) and the present study revealed that our model predicts more tundra and less steppe/desert than the previous reconstruction during LGM, and the estimated peatland growth is ~100 Pg C lower than the previous estimate. These two factors account for most of this difference. But we also note that this study includes a spatial domain that stretches further south and includes different depth intervals of soil (we exclude 2- to 3-m soil depth due to limited data for these soils outside the permafrost biomes).

## DISCUSSION

### Deglacial losses of land C

Despite a modest gross increase of ~60 Pg C from LGM to PI, there is a large reconstructed variability in C stocks over the course of deglaciation (Fig. 3), with a pronounced minimum around 11 ka (−340 Pg C relative to LGM). To further examine these large dynamic shifts in terrestrial C over time, we calculated net changes in the total stock between each millennium (Fig. 4A). The net change at each millennial time step is the sum of changes in land C stock from mineral soils, deep loess deposits, peatlands, and loss of subglacial C from beneath cold based ice. We also include an estimate of C which may have been remobilized and cycled via the atmosphere due to inundation, although we conservatively suggest that inundation preserved C stocks. We lack a precise estimate of how much of the inundated soil C may have been redistributed within the shelf sea system to eventually reach the atmosphere, but we speculate that it could be around 30% (range, 0 to 66%). This may be an underestimate; 66% of the soil C from eroded Yedoma deposits along the coastline of the Siberian Arctic is thought to reach the atmosphere (*36*). It may also be an overestimate; short term (<1 year) C losses of <10% from degrading and inundated soils have been reported for the circum-Arctic and permafrost coasts (*37*, *38*). Despite these uncertainties, we include this speculative estimate of 30%. The rationale behind the inclusion of inundated shelf soil C to our reconstruction is that modern coastal erosion displaces soil carbon, and past coastal erosion with rising sea levels should have been substantial. We choose to include this highly uncertain estimate rather than overlook its possible influence on the atmospheric composition.

We reconstruct substantial dynamic changes to loess C stocks. The first few millennia after the LGM saw net sequestration of C in loess due to continued deposition of windblown materials onto existing loess deposits. After 17 ka, warming and thaw accelerated the loss of C, coinciding with a pronounced rise in atmospheric CO_2_ during the 16.3-ka event (Fig. 4C) (*9*). This rapid rise has been attributed to increased ocean upwelling (*39*), but we reconstruct a terrestrial net release of 65 ± 113 Pg C from 17 to 16 ka, primarily caused by permafrost thaw of deep loess deposits (42 ± 71 Pg C). However, this C loss is largely dependent on the presence of a continuous permafrost zone in the North American loess sector that is affected by early thaw. This thaw of North American loess was only reconstructed when using TraCE-21k climate forcing but not with HadCM3 forcing. Observational paleo-evidence for extensive permafrost in this area is inconclusive (*40*), although records of polygonal terrain and ice/sand wedges have been found near the limit of the Laurentide ice sheet in Iowa and Nebraska during the LGM (*41*, *42*).

The loss of deep loess C continues until the beginning of the Holocene, with marked losses before 14 ka (49 ± 70 Pg C) and 11 ka (88 ± 61 Pg C), where the latter is influenced by thaw of the Central Siberian loess sector. Such rapid collapse of frozen loess-like deposits (Yedoma) in response to warming appears possible, as it occurs today (*17*). This rapid type of collapse in the form of thermokarst formation has been linked to high rates of CH_4_ release (*43*). It is therefore tempting to speculate that a connection may exist between the thaw of loess and the CH_4_ records from these two periods (*9*). However, others have noted that thermokarst lake formation within the Yedoma region occurred after the spikes in CH_4_ release seen at 14.8 and 11.7 ka (Fig. 4B) (*44*). We note that the study did not include records from the Central Siberian loess sector, which is of interest here. Another constraint that argues against CH_4_ release from permafrost thaw comes from the isotopic records of CH_4_ (*45*, *46*). Although we cannot resolve decadal- to centennial-scale rates of C release, we estimate that it is possible to reconcile the millennial rates with these isotopic constraints (<19 Tg CH_4_ per year), assuming that not all C was mobilized as CH_4_. Modern estimates from permafrost regions suggest that up to 10% of the C loss may be derived from CH_4_ (*47*). Last, we acknowledge that no elevated CH_4_ concentrations have been observed for 16 or 15 ka when our estimates also show large net C losses (Fig. 4, A and B).

These 14.8- and 11.7-ka events (*10*) are both periods of pronounced warming and relatively rapid sea level rise, and our results indicate that as much as 89 ± 171 Pg C was released from land between 15 and 14 ka and 127 ± 149 Pg C between 12 and 11 ka. Between 12 and 11 ka, the overall net carbon losses (excluding gains into peatlands) were primarily from loess (63%), mineral soils (20%), inundation (9%), and subglacial carbon storage (8%). As we force the permafrost reconstruction with additional temperature changes added to the modeled climate during this period, to better fit the permafrost extent with empirical data (fig. S2), we may possibly exaggerate the pace of this loss. However, our estimates do not include a proposed degradation of putative C-rich ice complexes on the Siberian shelf during these periods (*43*), which would further add to these C stock losses.

Our estimate of inundation losses of 30% (from atmospheric cycling of C) means that 12 ± 6 Pg C was released between 15 and 14 ka and 13 ± 8 Pg C between 12 and 11 ka. As we count these losses toward net changes, but not stock changes (Fig. 3), cumulative losses from land (including losses from inundated shelves) continue past the stock minimum at 11 to 10 ka. Adding these to the cumulative losses result in a release of ~440 Pg C of primarily old, carbon-14 depleted C (Fig. 4E). We do not propose that all this C remained in the atmosphere as the ocean, and tropical ecosystems, would have acted as a buffer. With a simple back of the envelope calculation based on a 25% airborne fraction over millennial scales (*48*, *49*), the atmospheric signal would amount to a total increase of 52 ppm between the 21 and 10 ka (Fig. 4). This is less than what is required to explain the atmospheric increase of ~80 ppm over this period. However, the contribution of land C to the overall atmospheric CO_2_ concentration change is dominant during certain millennia and even exceeds the atmospheric signal during 14 ka (Fig. 4). There could be several possible explanations for this behavior at 14 ka, such as uptake of C in other land regions, or an overestimation of C losses from our own region of interest. We note that 25% airborne fraction also rely on land C uptake, which may imply that the fraction could be higher as land C change is constrained across much of the land mass in our study.

### Holocene uptake

During the Holocene, there is a sustained net sequestration of C (Fig. 4, A and F), primarily not only into peatlands but also, to varying degrees, into mineral soils. This growth buffers losses from beneath cold based ice sheets which persist until 5 ka. The sink strength of peatlands is most pronounced during the early Holocene with a maximum increase of 61 ± 41 Pg C at 8 ka and 61 ± 47 Pg C at 7 ka. The cumulative gain during the Holocene amounts to ~370 Pg C (Fig. 4E). On the basis of a modeled atmospheric CO_2_ response of peatland C uptake (*50*), we calculate a decrease of ~23 ppm CO_2_ from a linear function, corresponding to ~13% uptake response. These results imply that other sources of atmospheric carbon must have been active during the Holocene to reconcile the atmospheric record. Constraints from the ocean δ^13^C record suggest that about 290 Pg C was incorporated into terrestrial storage during the early Holocene, with concurrent release from the oceanic storage in response to this uptake (*51*). Later in the Holocene, the same δ^13^C records show a moderate net release of terrestrial C into the atmosphere.

It is important to acknowledge that the proposed stock changes in Pg C are massive. However, these changes are derived for millennia. Yearly changes (table S7) are comparatively smaller than what is now estimated as a yearly sink due to land use globally (2 Pg C) (*52*).

### Land as a source and sink

Our results clearly demonstrate that northern land masses played an important role in the deglacial atmospheric C dynamics. Although the uncertainty is high, and the results primarily rely on using modern analogs for past C storage, they strongly indicate substantial C transfers between systems. They corroborate recent insights that the ocean did not act alone as a C source during the deglaciation (*4*, *53*, *54*) and add quantification and timing of changes in land C stock sizes. The reconstructed changes in soil C stocks are large, abrupt, and coincide with major atmospheric events. We also posit that we may still underestimate the importance of land C dynamics in the deglacial C cycle. We do not account for other important land features south of 23°N, such as the Green Sahara (*55*), tropical rainforests (*56*), tropical wetlands (*28*, *57*), and the Sunda shelf in South-East Asia (*14*, *58*). We believe that further study of these systems is needed to fully explore the role of the land surface as both a sink and a source of C during the course of deglaciation.

Our findings stress the importance of these large-scale system changes as they resulted in the transfer of C measured in billions of tons (Pg C), which may have either augmented or depressed atmospheric CO_2_ levels, depending on the specific processes at play, e.g., abrupt permafrost thaw or peatland growth. Thus, the timing of these events may have worked to partly conceal the total gain or loss of C tied to each of these processes and their potential impact on the atmospheric CO_2_ level. The convoluted signal in the deglacial atmospheric δ^13^C record (*59*) may also be a testament of this, as the signal responds to release of terrestrial C. We find it possible to reconcile the hypothesis of a dominant Southern Ocean to explain C dynamics over glacial to interglacial scales with a responsive and variable terrestrial biosphere, which would act on shorter timescales as both a sink and a source. It remains to be explored whether the contribution from land had any lasting impact on the atmospheric CO_2_ rise and the isotopic signals, and what that in turn might reveal of the future resilience of the Earth system.

## MATERIALS AND METHODS

### Climate models

For our biome reconstructions, we used six climate variables from two independent climate model simulations, the Community Climate System Model version 3 (CCSM3) TraCE-21k (*60*), and from HadCM3 [see Data and materials availability to access these data (*61*)].

The TraCE-21k simulation consists of transient climate evolution for the past 21,000 years (*60*, *62*). TraCE-21k was set up using the National Center for Atmospheric Research’s CCSM3 with time-evolving boundary conditions including orbital parameters (*63*), greenhouse gas concentrations (*64*), melting water discharge (*65*), and continental ice sheets (*66*). TraCE-21k is a fully coupled (with dynamic atmosphere, ocean, sea ice, and land modules) nonaccelerated transient climate model simulation from the LGM to present day. All climate variables from this simulation are regridded from the original ~3.75° by 3.75° atmosphere/land model to a 1° by 1° resolution with bilinear interpolation. Although this transient dataset offers details of many aspects of the deglacial climate evolution (*67*–*70*), we averaged the climate over 300 years before each millennium to produce comparable estimates to the HadCM3 simulation.

The HadCM3 data are from time-slice experiments spanning the last 21 ka to present in 1 kyr steps, modeled using an ensemble from the UK Met Office HadCM3 General Circulation Model with a resolution of 3.75° by 2.5°, which was regridded to a 1° by 1° resolution with bilinear interpolation. HadCM3 consists of a linked atmosphere, ocean, and sea ice model, can be run for multimillennial length simulations, and has been widely used to study past, present, and future climates. The model does not include interactive ice sheets, carbon cycle, or methane. Any changes in orbit, greenhouse gases, dust, ozone, and ice sheet evolution must be prescribed. The prescribed boundary conditions for each model integration are outlined in table S9. The PI simulation was set up following control guidelines from the Palaeoclimate Model Intercomparison Project. For paleo-changes to planetary orbit, greenhouse gas concentrations, and ice sheets, the boundary forcing applied by Singarayer and Valdes (*71*) was used (see table S9 for details). The LGM ice sheet configuration is consistent with that of Trace-21k (*66*). Each ice sheet configuration was linearly interpolated onto the resolution of the GCM grid.

The climate input for the biome reconstructions was restricted to mean annual air temperature at 2 m height, the temperature of the warmest month (July) and the coldest month (February), as well as the temperature range between those two months, total annual precipitation, and lastly the growing season precipitation, here defined as the precipitation occurring during months with temperatures of >5°C. Many of these variables were highlighted in a method study of biome reconstructions with machine learning (*72*).

### Biome reconstructions

Biome areas were modeled by a machine learning algorithm [Random Forest from ScikitLearn; (*73*), following the methods described in (*74*)]. Given training data of biomized pollen (*75*–*78*) paired with a subset of modeled climate parameters (HadCM3 and TraCE-21k climate forcing) and topography (*66*), the algorithm (*79*) predicted the most probable distribution of biome zones across the northern land surface grid. This data-driven approach decreases the impact of potential systematic errors in the climate input (*74*). The biome classes were restricted to tundra, boreal forest, temperate forest, warm-temperate forest, tropical forest, grassland/dry shrubland, savanna/dry woodland, and desert. These correspond to the mega biome classes defined within the Biome6000 project (*77*). This required translation of the biome nomenclature in the dataset from North America (*76*). We were able to translate most of the sites with good confidence (>80% match) by pairing overlapping interpretations between this dataset and the Biome 6000 dataset (*77*) at 0 ka.

The pollen datasets were allowed to overlap and contain duplicates to permit statistical spread where interpretations diverge or stronger statistics for sites where several samples reveal the same biome. However, we found instances of interpretations which were not allowed to remain. These indicated tundra in areas where the mean annual temperature was >5°C in the climate datasets. Most of these likely erroneous interpretations of a cold tundra biome were located in Central or Southern Europe at low altitude during the Holocene, and we believe that the proper interpretation may have been a heath-like landscape or possibly wetlands. We did not remove interpretations of tundra from the Biome6000 dataset.

To perform a better prediction, we separated the training data into two separate periods: the climatically stable Holocene (9 to 0 ka; 15,610 training points) and the climatically dynamic deglaciation (21 to 10 ka; 7,164 training points). For each millennium, the accuracy of prediction was 80% on average and never lower than 70% (table S8).

Because of the shortage of desert evidence in the pollen data, we added sites at 0 ka in areas known as deserts today (242 locations, randomly selected using ArcMap v. 10.6). We also added all desert locations from the 6-ka pollen data to the deglacial run, as well as 40 locations randomly selected in areas recognized as desert at 21 ka based on a previous paleo-reconstruction (*80*).

### Mineral soil C

For each reconstructed 1-kyr time slice, storage of soil C in mineral soils (0- to 2-m depth) was calculated per 1° resolution grid cell based on the probability of a biome being present (*81*). To reach this probability, the algorithm counts the fraction of decision trees in the forest that vote for a certain class. We translate this biome probability to an areal fraction of the cell, thereby allowing several biomes to occupy a single cell. The areas were then multiplied with the typical biome-specific soil C stocks (carbon transfer functions; table S1). The sum of these results details the stock per cell. We assume soil C stocks to be in equilibrium with their biome within a millennium. Carbon transfer functions for biomes outside the permafrost region were calculated by combining gridded soil C data (*82*) with modern biomes (*26*). For those areas which were underlain by permafrost, transfer functions were estimated similarly to methods in the study of Lindgren *et al.* (*13*), using the Northern Circumpolar Soil Carbon Database (*24*) and data from the Tibetan Plateau (*25*), together with modern biomes (*26*). In short, we calculated averages of kilograms of carbon per square meter (transfer functions) normalized by polygon area for each separate biome, with and without permafrost.

For each time slice, changes in soil area were calculated on the basis of sea level rise and ice sheet isostacy (*66*), as well as ice sheet boundaries adjusted to 1° resolution (*30*, *31*). The soil areas were also corrected to represent only mineral soils by removing the percentage covered by peat in each cell (see the section about peat). Steep slopes (>4° slope) were also calculated separately as we do not assume the C content of these soils to be biome dependent, consistent with the methodology in the study of Lindgren *et al.* (*13*). Slope areas were identified using the mean slope product of ISLSCP II HYDRO1k (*83*) with 0.5° resolution. The finer resolution allowed us to calculate a fraction of a 1° cell as steep. This fraction of area was multiplied with a carbon transfer function of 3 kg m^−2^ for alpine C stocks (*84*).

We calculated our mean mineral C stock as the average of the C stocks derived from the two different climate models. The C stock differences between the two models were relatively small in comparison to the overall uncertainty of the mineral C stock based on SDs (2 to 16% of the SD).

### Permafrost extent

Permafrost affects soil C storage, and we modeled its extent for each millennium as a function of mean annual temperature after the study of Chadburn *et al.* (*85*)*.* We restricted the reconstruction by using the model’s 100% coverage as indicative of continuous permafrost, which gave the best fit compared to observed permafrost extent (*86*). As this equation needs absolute temperatures and is sensitive to any systematic bias in the climate data, contrary to our biome reconstruction methodology, we adjusted the modeled mean annual temperatures to fit our results with empirically estimated permafrost extents for present day (*86*), Mid-Holocene (*87*, *88*), and the LGM (fig. S2) (*40*). For the HadCM3 mean annual temperature, we applied an adjustment of −1°C per millennium between 10 and 17 ka, meaning that we progressively increased the temperature difference between the present and LGM. This allowed permafrost to reach the reconstructed extent in Europe at the time of the LGM, which is well constrained by evidence (*40*), see fig. S2 for a comparison between modeled and reconstructed permafrost boundaries. For the TraCE-21k mean annual temperature, we first applied a +5°C adjustment for all millennia to fit the permafrost distribution at present day. We then applied a similar adjustment to the one done for HadCM3 between 10 and 17 ka. This resulted in a slight underestimation of the LGM permafrost area in Europe for the TraCE-21k data but a larger extent in North America, south of the Laurentide ice sheet, a region from which there is no conclusive evidence for widespread continuous permafrost at the LGM.

As we reconstruct permafrost for the two climate models separately, we end up with two delineations. Because the estimated mineral soil C stock for each 1-ka time slice (not including peat C or loess C) is averaged from the two model runs, the effect of diverging permafrost estimates is limited. For loess deposits, we average the area affected by thaw between the two models and use the divergence between the two permafrost delineations in the calculated uncertainty ranges. While the difference in permafrost extent between the HadCM3 and TraCE-21k models has limited impact on estimated mineral soil C and peat C stocks, it has important implications for putative C losses from deep loess deposits in the early stages of deglaciation (see below).

### Loess extent, depths, and C stocks

Areas of loess deposits within our domain are located in Central Europe, Central Siberia, the United States, and China. The methodology to map these areas and other details of the ~50 loess sections used to derive soil C data for this analysis are detailed in the supplementary of the study of Lindgren *et al.* (*13*).

Following Lindgren *et al.* (*13*), we reconstruct C storage in deep loess deposits (>2 m) as being much larger under permafrost conditions, comparable to Yedoma deposits, than in thawed-out deposits. As loess deposits thaw in our reconstruction, they lose C. However, we do not assume that all loess C in the former permafrost regions is lost. From an estimated 363 Pg C at 21 ka (and 398 Pg C at 17 ka), we calculate that 57 Pg C remain today. This calculation is largely based on a marked decrease in average %C in the thawed-out loess deposits compared to intact Yedoma loess deposits.

We assume that these losses occur within the millennium of permafrost thaw. We consider this a reasonable simplification based on two datasets. On the one hand, we have very large C losses in loess C since postglacial thawing (sometime between 9 and 7 ka), as described above. On the other hand, Elberling *et al.* (*89*) described a very fast C loss in a 12+ year long incubation experiment (aerobic, 5°C) of aeolian deposits in NE Greenland following thaw, with cumulative losses surpassing 50% over that time period. Combining these two datasets suggests >90% C loss within a millennium, with only a small fraction of a very passive C pool decaying over much longer timescales.

The timing and patterns of this loess C loss in our reconstruction are determined by delineations of the permafrost extent between the 1-kyr time slices, overlain with the spatial location of the loess deposits. In our integrated assessment, we provide the mean extent of permafrost in loess at millennial time steps, based on the maximum and minimum areas using two climate models. Uncertainty estimates have been calculated based on SD of loess deposit permafrost area and depth per region (US, Europe, Central Siberia, and China) and propagated for the full stock in Pg C.

We calculated changes in C stocks of loess deposits at millennial time steps for each loess region (US, Europe, Central Siberia, and China). An important consideration is the quality of the chronological control available in each of the ~50 loess sections considered. Even in the few cases in which multiple radiocarbon [or optically simulated luminescence (OSL)] dates were available, they often came with large uncertainty ranges or even inverted ages. We, therefore, aimed to identify the most likely depths of the 21, 18, 15, and 12 ka isochrones in the loess sections and then interpolated to millennial time steps to conform with the temporal resolution of the biome shifts and ice sheet declines.

We consider accumulation of C during the 21 to 17 ka period, as there was a negligible loss of permafrost area, but loess deposition continued resulting in the additional storage of C through syngenetic permafrost aggradation. There is good radiocarbon-dated evidence for continued deposition at multiple sites in all regions (*90*–*93*). See fig. S3 for temporal evolution of loess soil C stocks.

### Peatlands

Our estimates of the net peatland development over time are based on curves from Loisel *et al.* (*94*) for peatland area (MGK13S curve) and Yu (*29*) for peat C stock accumulation, which are based on modeled net carbon balances. We refer to those references for more information on these data. To estimate the present (PI) peatland extent and C stocks, we use maps of peatland extent presented in the work of Hugelius *et al.* (*95*). We estimate the total PI peatland C stock to 450 Pg C (*95*), which is higher than the current stock since an estimated 30 to 40 Pg peatland C has been lost because of peatland drainage (*95*, *96*). Using curves of vertical peat C accumulation rates over time from Yu (*29*), the peatland carbon stocks for each millennium were calculated as a fraction of the total PI peatland C stock.

When a peatland expands, the area of mineral soil needs to be adjusted to avoid double counting. To account for peatland expansion across other mineral soils types, we use the MGK13G dataset (oldest peat initiation date per grid cell) (*94*) to identify all grid cells with known peatland extent per millennium. This analysis is carried out at a 50-km resolution in an equal area grid (the MGK13G dataset was interpolated from 1° resolution). For areas south of latitude 38°N (0.7 million km^2^ of the total 3.7 million km^2^ peatland area), there was no MGK13G data, and peatland initiation of 12 ka was assumed (based on the pattern observed in 40° to 38°N). For each millennium, the cumulative growth of peatland area (*94*) is distributed to grid cells with known peatland occurrence (as determined from the interpolated MGK13G and peatland extent maps), proportionally to the PI peatland extent in that grid cell. In this way, peatland growth is modeled proportionally to total increase of peatland area and the present-day coverage in each cell, but it does not saturate (i.e., reach the full PI extent) until the most recent century. In addition to present-day uncertainties in estimates (±10% for extent and ±36% for Pg C stocks), we assume that the modeling back in time adds an uncertainty of ±50% at 16 ka which decreases linearly to 0% at the PI. These two sources of uncertainty are combined using additive error propagation.

### Carbon in formerly glaciated areas

Formerly glaciated areas started accumulating C as soils developed (*32*). To estimate how much carbon these areas sequestered, we applied the equations developed by Harden *et al.* (*32*). These equations are soil specific, but we interpreted them to represent biome types: spodsol for boreal forest, cryosol for tundra, and mollisol for grassland and dry shrublands. We adjusted the carbon equilibrium constant (*Ce*) for each equation, applying our average C transfer functions (kilograms of carbon per square meter) for each biome type rather than the predetermined constants (which were lower in the original reference, perhaps due to a lower soil depth in that analysis). Separate *Ce* were used for the 0 to 1 m and 1 to 2 m, again based on our estimated C transfer functions. We allowed the carbon to build up according to these equations over three millennia. This may not be enough time to reach full C storage capacity for grassland and dry shrublands, but it is sufficiently close for boreal forest and tundra. After three millennia, these soils were transferred to the biome-based calculations for mineral soils.

The ice sheets retreat following previously published delineations (*30*, *31*), adjusted to 1° resolution. The ice sheets from North America were given in radiocarbon years. We applied the IntCal13 calibration curve (*97*) to translate these into calendar years. Although the dataset from North America contained ice-dammed lakes, these have not been included in the analysis; instead, these areas are considered as soils in our analysis. We justify this by the lack of data for ice dammed lakes from Europe. The only lake which is not considered soil or land is the Caspian Sea (*98*).

### Carbon beneath cold based ice sheets

Cold-based ice sheets have low erosional impact (*99*) and may preserve preglacial landscapes and soils; such soils exist beneath the Greenland ice sheet today (*100*). We include a hypothetical subglacial high-arctic tundra soil C stock (*101*) down to 2 m in cold based areas. The delineation of cold-based ice follows (*102*). We also include additional C in peat down to 1 m across 1% of the area (*13*). This stock is removed as the ice sheets retreat; present-day observations of C fluxes from retreating ice sheets support rapid oxidation (*103*). However, simultaneously, new soil C begins to build up according to the method described above. These two different C stocks are reported separately.

### Carbon on exposed sea shelves

The mineral soil C on exposed sea shelves is calculated on the basis of changes in exposed soil area on what is present-day sea shelves. There is no specific calculation for these areas; instead, they are treated similarly to the mineral soil C on land. The area of the exposed shelf is calculated as the difference between the land area at each time step and the land area at present. The changing shoreline is determined at 1° resolution (*66*). Peat soils and deep loess deposits are not considered on the shelf.

### Carbon on inundated sea shelves

The mineral soil C on inundated sea shelves is estimated on the basis of the loss of area from one millennium to the next as sea level rises. The soil C which was present on land at time *t* is then transferred to inundated C at time *t* + 1 if calculated from 21 to 0.

We estimate the storage of C on the inundated shelf at present to be higher than the storage on the fully exposed shelf at the LGM. This means that carbon storage on the exposed shelves increased before inundation.

At the time of inundation, some of the soil C is assumed to cycle via the atmosphere when we estimate net changes between millennia. But this atmospheric cycling is not reflected in the total C stock as we assume losses from cycling to be compensated by deposition of both marine and terrestrial C on the sea floor. We speculate that the 30% (0 to 66%) of the inundated C is cycled with the atmosphere (*36*–*38*). This number is highly uncertain for several reasons. Eroded soil organic C can be stabilized in association to heavier mineral particles and may settle rapidly offshore due to ballasting (*104*, *105*). Depending on the depositional environment offshore, C can be held in suspension, buried on the shelf, or transported toward the continental slope on millennial timescales (*36*, *106*, *107*). The long-term fate of soil C once it reaches the ocean remains unclear, and there is no current estimate of the full influence of coastal erosion on atmospheric CO_2_.

Reaching a firm estimate of how inundation or erosion affects C cycling will be challenging. For the Arctic coast, where most of the past sea shelf inundation occurred, CO_2_ release depends on multiple factors which are poorly constrained. This includes the initial soil C content and quality, the availability of oxygen (*108*, *109*), the presence of thermoerosional processes or thermokarst lake formation before actual erosion (*17*, *110*), the lengths of the open-water season and residence time of eroded material on land before transport into the ocean (*38*, *111*), as well as the depositional conditions and production rates on the shelf (*106*, *107*, *112*), CO_2_ pathways within the seawater column (*113*–*115*), and marine primary production (*116*). Some of these factors reveal that we may risk a double accounting of CO_2_ losses. Any measurements of CO_2_ in the coastal zone that are related to processes on land, or oxidation of soil C which has been transferred laterally through the landscape, should already be accounted for in our other estimates. Our cycling estimate should only reflect the fate of the in situ soil C as it is inundated.

### Gross changes

We calculated the gross changes between different millennia by using maps of C content (see table S2). Explicit C content in each raster cell was thus compared for two millennia and then summarized. By looking at total gains of C and total losses of C, rather than comparing net changes, we can better understand how the C was shifted geographically from the LGM onward. We have not processed how this may have affected the atmosphere but note that any temporal delays between the gains and losses across a millennium (which we cannot compute with our time resolution) could have affected the atmospheric CO_2_ record.

### Uncertainty estimates

Uncertainty estimates are generally based on SDs (unless otherwise specified). These uncertainties have been propagated for the total stock with the square root of summed variances. For the net changes, no obvious method of error estimation was available. Because of large stock sizes and errors compared to relatively small changes between millennia, it was not informative to propagate errors for the stocks at two separate times, nor did it seem reasonable as an overestimation in stock at time *t* would, most likely, also be an overestimate at time *t* + 1. Instead, we related the variance ( $\sigma^{2}$ ) in stock to the actual mean stock size (μ) , resulting in a coefficient of variation (*V*; Eq. 1) which can be used to approximate uncertainty of the estimate ( $E_{∆x}$; Eq. 2), where *V* is averaged over two consecutive millennia ( $V_{t}\;$and $V_{t+1}$ ) and multiplied with the change in stock size ( ∆).

$$V=\frac{\sigma^{2}}{\mu }$$ (1)

$$E_{∆}=\sqrt{\frac{\left[V_{t}+V_{\left(t+1\right)}\right]}{2}\times |\;∆\;|}$$ (2)

We propagate the combined uncertainty estimates over all soil C stock changes $E_{∆\text{mineral}}$ , $E_{∆\text{peat}}$ , etc. by the square root of summed variances.

### Atmospheric CO_2_ concentrations

To relate our net transfers of C to a possible atmospheric CO_2_ impact, we translated our estimates of Pg C to atmospheric CO_2_ (parts per million) assuming a constant mass of the atmosphere over time (5.15 × 10^21^ g). As not all of the released C will remain in the atmosphere due to ocean uptake, we used an estimate of airborne fraction: 25% (*48*) which falls within a range of estimates reviewed in (*49*). As this estimate represents the atmospheric response to a C loss, we relied on another modeling study to look at the response of land uptake for our peat accumulation (*50*). In this study, the authors had used a box model (*10*) to study the atmospheric CO_2_ response to global peatland C uptake over the deglaciation and Holocene. We did not use the model itself but instead relied on the near linear relationship between peatland C uptake and CO_2_ decrease over the relevant time period. This linear relationship indicated an uptake response of ~13%. Error bars indicate the maximum or minimum contribution of CO_2_ based on the errors estimated for the net C transfers. We compare the CO_2_ changes from our results with ice core records of atmospheric CO_2_ (*117*), calculated here as the net sum of differences between all available data points within each millennium.

## Supplementary Materials

### This PDF file includes:

Figs. S1 to S3 Tables S1 to S9 References
