# Supplementary material for: Massive losses and gains of northern land carbon stocks since the Last Glacial Maximum
Source: Sci Adv. 2025 Aug 29;11(35):eadt6231. doi: 10.1126/sciadv.adt6231 (PMC12396326; doi:10.1126/sciadv.adt6231)
Supplement: Supplementary file 1 — Figs. S1 to S3 Tables S1 to S9 References [file sciadv.adt6231_sm.pdf]

Science Advances
AAAS

Supplementary Materials for

Massive losses and gains of northern land carbon stocks since the Last Glacial Maximum

Amelie Lindgren *et al.*

Corresponding author: Amelie Lindgren, amelie.lindgren@gu.se

Sci. Adv. **11**, eadt6231 (2025)
DOI: 10.1126/sciadv.adt6231

This PDF file includes:

Figs. S1 to S3
Tables S1 to S9
References

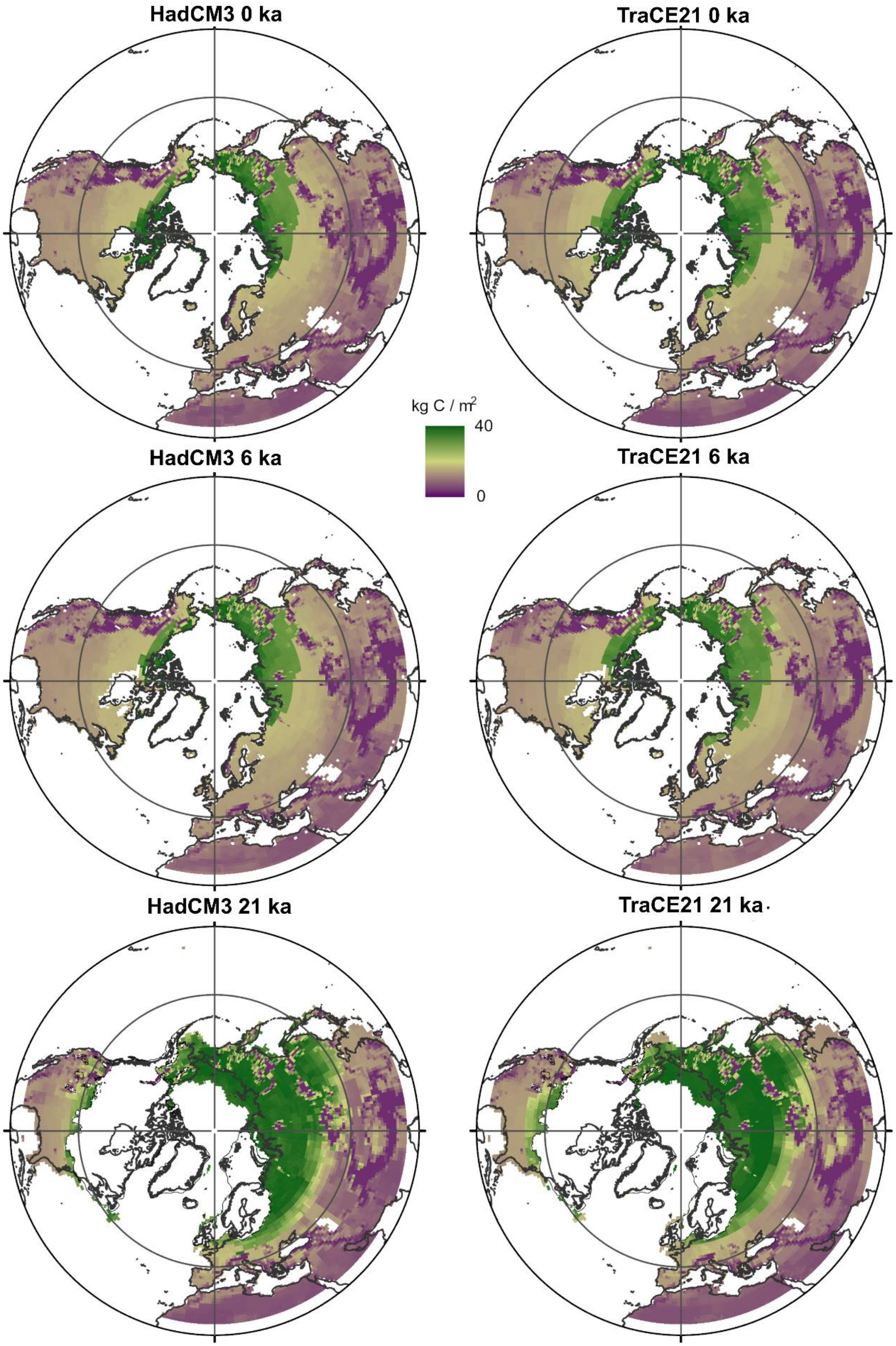

Fig. S1. Geographical distribution of mineral soil C

Mineral soil C storage in kg m^{-2} at 0 ka, 6 ka and 21 ka as reconstructed from climate-biome modeling for two separate climate models: HadCM3 and TraCE-21k. Note that steep areas are treated separately from the climate-biome modeling.

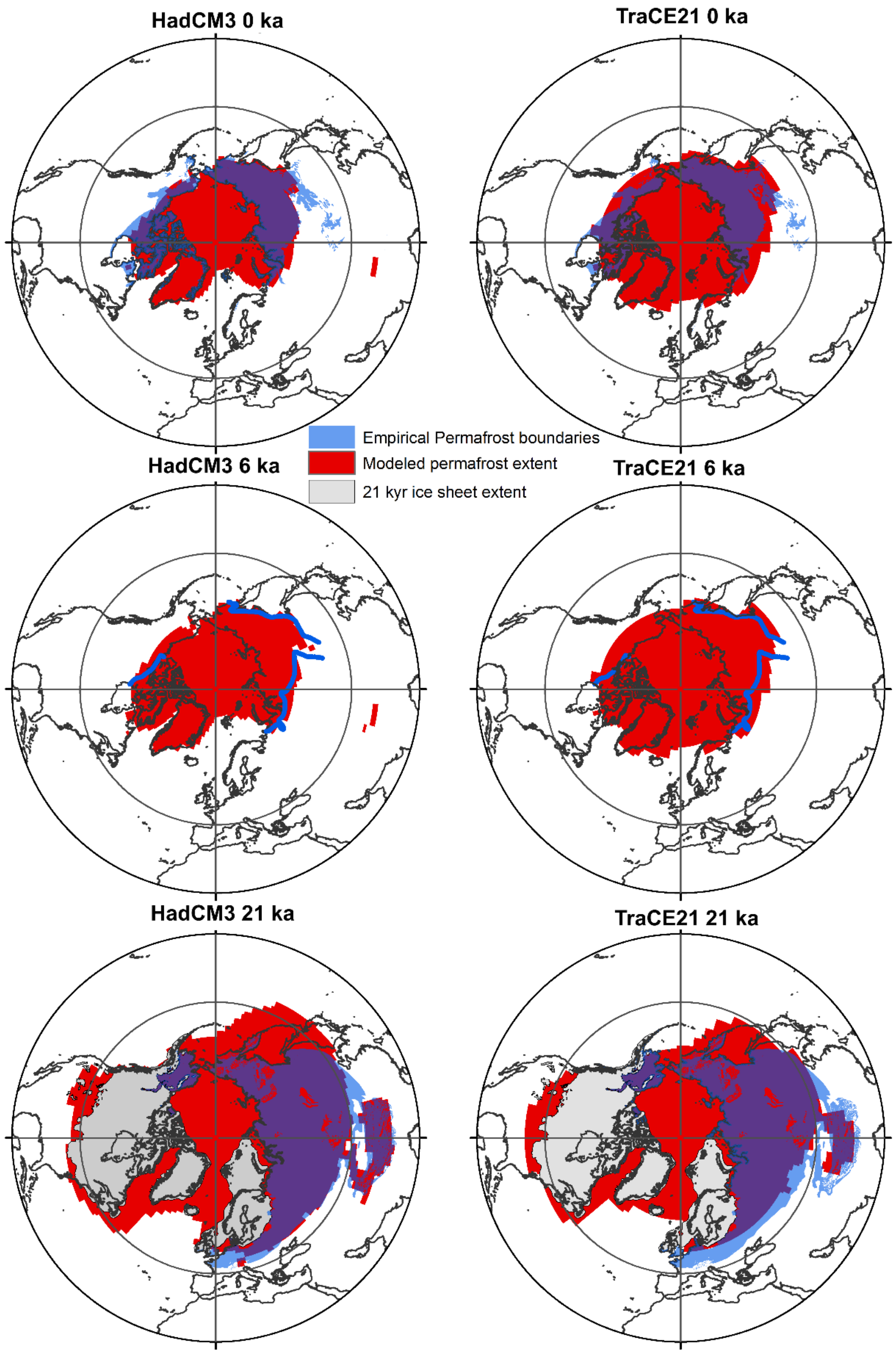

Fig. S2. Geographical distribution of permafrost

Comparison of our modeled (red) and empirically reconstructed continuous permafrost extent (blue). The modern permafrost is after (*86*), the mid-Holocene extent (blue lines) is from (*87*, *88*), and the LGM permafrost extent is from (*40*).

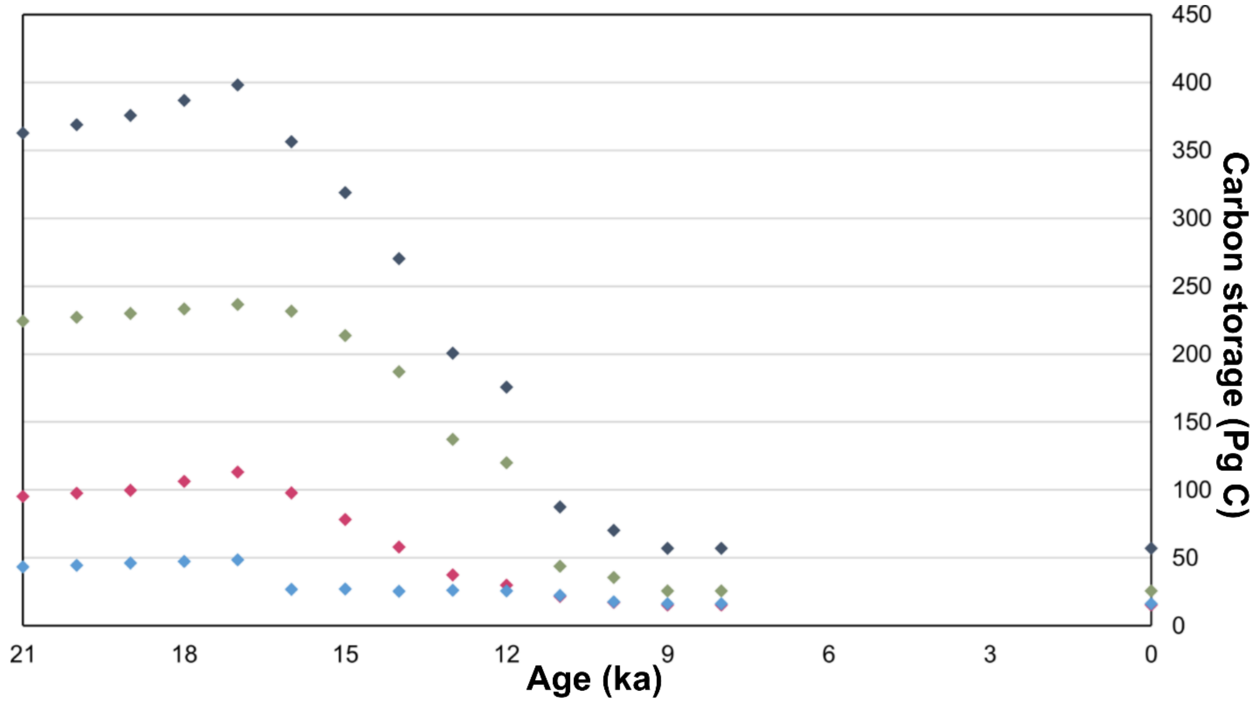

Fig. S3. Storage of C in loess deposits

Total C storage in loess (north of 23 ˚N) per millennium from 21 ka until 0 ka in Pg C. We report the total stock (dark blue) and by sectors: North America (light blue), Europe (Pink), Asia (Green).

Original Biome Nomenclature	Biome nomenclature (this study)	0-1m kg C m^{-2}	SD kg C m^{-2}	1-2m kg C m^{-2}	SD kg C m^{-2}
Non-Permafrost Biomes					
Boreal Forests/Taiga	Boreal Forest	13.8	6.2	2.9	2.6
Tundra	Tundra	13.1	4.1	6.3	5.7
Temperate Broadleaf and Mixed Forests	Temperate Forest	10.8	4.8	3.3	2.4
Tropical and Subtropical Forests	Subtropical Forest; Warm-Temperate Forest	9.1	4.6	3.5	2.5
Temperate Grasslands, Savannas, and Shrublands	Grassland and Dry Shrubland; Savanna and Dry Woodland	10.4	4.6	2.8	1.4
Deserts and Xeric Shrublands	Desert	4	2.4	1.6	1.8
Permafrost Biomes					
Boreal Forests/Taiga	Boreal Forest	20.2	17.4	10.1	9.1
Tundra	Tundra	26.7	15.9	12.8	11.6
Deserts and Xeric Shrublands	Desert	4	2.4	1.6	1.8
Alpine Meadow	Grassland and Dry Shrubland	7.5	4.8	3.1	4.5

Table S1. Soil carbon in different biomes

Summary of biome nomenclature as well as estimated typical soil C stocks per biome type. These carbon transfer functions are reported per areal unit (kg C m^{-2}) with one standard deviation (SD) for mineral soils, categorized by biome and soil depth. These have been calculated as weighed averages of soil C stocks in kg m^{-2} from two databases of modern C stocks, see (*13*).

Age (ka)	Gross increase (Pg C)	Gross decrease (Pg C)	Difference (Pg C)	Percent decrease of difference
0	9.0	-11.6	-2.6	446%
1	8.6	-11.5	-2.9	400%
2	19.7	-9.9	9.9	-100%
3	16.8	-12.9	3.9	-332%
4	14.4	-13.6	0.8	-1632%
5	23.4	-11.9	11.5	-104%
6	30.6	-13.5	17.1	-79%
7	31.6	-24.0	7.6	-314%
8	44.9	-18.3	26.7	-69%
9	68.3	-48.8	19.5	-250%
10	31.6	-40.3	-8.7	464%
11	33.4	-63.9	-30.5	209%
12	32.8	-22.0	10.8	-204%
13	47.8	-40.3	7.5	-537%
14	34.8	-65.1	-30.2	215%
15	34.9	-59.4	-24.6	242%
16	38.1	-51.8	-13.7	378%
17	38.7	-28.3	10.4	-273%
18	27.2	-17.0	10.1	-168%
19	12.7	-16.3	-3.6	458%
20	12.9	-17.0	-4.2	410%

Table S2. Gross changes of mineral soil C calculated per grid cell.

Gross increase and decrease of mineral soil C (Pg C) due to geographical shifts of C between millennia. The C which is lost due to inundation is removed from the gross decrease as this C is rather moved to another storage unit (inundated C).

Age (ka)	Mineral Soil C storage TraCE 21 (Pg C)	Mineral Soil C storage HadCM3 (Pg C)	Mean soil C storage (land) (Pg C)	Standard deviation of mean soil C storage (Pg C)	Steep soil C (Pg C)
0	986	940	995	369	32
1	990	941	998	368	32
2	995	942	1001	372	32
3	976	941	991	362	32
4	980	929	987	355	32
5	976	933	987	360	32
6	964	923	976	360	32
7	951	904	959	351	32
8	951	890	952	360	32
9	917	881	930	352	30
10	898	871	915	377	30
11	911	885	928	427	30
12	937	915	956	503	30
13	918	920	948	518	29
14	916	925	949	557	28
15	957	943	978	622	28
16	989	972	1009	676	28
17	1011	994	1031	741	28
18	993	1004	1027	734	28
19	994	1005	1028	734	28
20	999	1013	1034	739	28
21	1003	1017	1038	743	29

Table S3. Soil C storage in mineral soils on land

Soil C storage in mineral soils across the domain at each millennium on land (Pg C) for the two separate model runs. The mean mineral soil C storage also includes the storage in steep areas, which is also presented separately. These estimates do not account for storage on the exposed sea shelves, and are thus constrained to the present day land mass. Standard deviation is presented for the mean mineral soil C stock on land (excluding steep areas).

Age (ka)	Loess stock average (Pg C)	Standard deviation (Pg C)
0	57	28
1	57	28
2	57	28
3	57	28
4	57	28
5	57	28
6	57	28
7	57	28
8	57	28
9	57	28
10	70	36
11	87	44
12	175	104
13	201	117
14	270	160
15	319	186
16	356	206
17	398	220
18	387	216
19	376	209
20	369	209
21	363	209

Table S4. Carbon storage in loess deposits

Millennial storage of C (Pg C) in loess deposits across the domain. C is lost through permafrost thaw.

Age (ka)	Cumulative stock (Pg C)	Change (Pg C)	Standard deviation (stock) (Pg C)
0	450	37	162
1	413	20	149
2	393	20	142
3	372	29	137
4	344	29	130
5	315	45	123
6	270	41	109
7	229	61	97
8	168	61	74
9	106	49	49
10	57	41	28
11	16	12	8
12	4	2	2
13	2	1	1
14	1	1	1
15	0	0	0
16	0	0	0
17	0	0	0
18	0	0	0
19	0	0	0
20	0	0	0
21	0	0	0

Table S5. Soil C accumulation in peat

Accumulation of peat soil C (Pg C) across the domain at millennial intervals with standard deviation given for the stock estimate.

Age (ka)	Soil C exposed shelf (Pg C)	Standard Deviation (shelf) (Pg C)	Inundated soil C (Pg C)	Standard deviation (inundated) (Pg C)	Cumulative inundated (Pg C)
0	0	0	0	0	263
1	0	0	1	0	263
2	1	0	2	1	262
3	3	1	2	1	260
4	5	2	4	2	258
5	9	4	5	3	254
6	14	6	4	2	248
7	17	8	7	4	244
8	23	11	37	23	237
9	54	33	31	22	201
10	80	52	26	19	170
11	101	68	44	32	144
12	147	107	18	10	100
13	162	116	16	10	83
14	168	118	34	25	67
15	203	148	12	7	33
16	209	154	2	1	21
17	202	151	10	8	19
18	205	153	2	1	9
19	196	144	6	6	8
20	200	149	1	1	1
21	201	151			

Table S6. Soil C storage on exposed and inundated sea shelves

Estimated changes of soil C (Pg C) on exposed sea shelves within the domain from biome changes and loss of land mass. Inundated C (Pg C) at each millennium is calculated from the C that was there during the previous millennium, and located within the geographical region which is inundated. Included is also the estimated cumulative storage of C on inundated shelves if all C is preserved or compensated by new sedimentation of C at the sea floor. Standard deviations are also given in Pg C.

Age (ka)	net-change Pg C kyr^{-1} Tg C yr^{-1}	propagated error (Pg C)	ppm CO_2 25%	ppm CO_2 uptake 13%	ppm CO_2 max release	ppm CO_2 max uptake
0	34,2	49,9		-2,09	1,84	-5,14
1	17,1	41,7		-1,05	2,71	-3,68
2	29,6	50,4		-1,81	2,20	-5,02
3	32,0	50,0		-1,95	1,90	-5,13
4	27,7	43,1		-1,69	1,26	-4,62
5	48,8	54,4		-2,98	-0,01	-6,66
6	53,2	58,0		-3,25	0,02	-7,12
7	48,2	58,3		-2,94	0,36	-6,97
8	52,8	63,1		-3,22	-1,79	-9,61
9	20,4	59,9		-1,25	0,74	-7,19
10	-10,7	88,8	1,25		8,82	-7,08
11	-126,6	149,1	14,83		29,11	-4,69
12	-24,5	76,2	2,87		10,13	-4,84
13	-74,7	92,8	8,74		17,20	-2,82
14	-88,9	171,2	10,41		28,53	-8,07
15	-71,9	142,0	8,42		24,56	-6,16
16	-64,5	113,3	7,55		20,50	-4,05
17	11,8	105,1		-0,72	10,04	-8,71
18	9,5	76,6		-0,58	7,04	-6,39
19	-1,3	78,7	0,08		9,11	-5,67
20	1,7	43,2		-0,10	5,12	-3,03

Table S7 Net transfers of land C at millennial scales

Net transfers of C storage (Pg C per ka or Tg C per year) across the domain with errors, as well as the possible atmospheric impact of CO_2 from the net transfers at millennial scales. Two options of atmospheric CO_2 contribution (ppm) are given based on different airborne fractions. Uptake responses are visible in a separate column. These numbers include the possible cycling of C (30%) from shelf inundation. Maximum release and uptake are estimated by using the errors of net C transfers. Release of CO_2 is always estimated with a 25% airborne fraction, and uptake is always estimated by 13% uptake response.

Age (ka)	Training data (ka)	Accuracy HadCM3	Accuracy TraCE-21k	n
**0**	0, 6, 9	86%	84%	182
**1**	0, 6, 9	83%	82%	172
**2**	0, 6, 9	70%	71%	184
**3**	0, 6, 9	77%	81%	180
**4**	0, 6, 9	77%	79%	185
**5**	0, 6, 9	81%	84%	177
**6**	0, 6, 9	88%	85%	169
**7**	0, 6, 9	80%	80%	185
**8**	0, 6, 9	81%	82%	180
**9**	0, 6, 9	82%	80%	176
**10**	9-21	81%	85%	181
**11**	9-21	72%	74%	190
**12**	9-21	79%	75%	174
**13**	9-21	76%	77%	184
**14**	9-21	74%	72%	176
**15**	9-21	80%	83%	181
**16**	9-21	79%	81%	177
**17**	9-21	83%	83%	179
**18**	9-21	80%	82%	142
**19**	9-21	81%	78%	116
**20**	9-21	86%	88%	106
**21**	9-21	74%	74%	195

Table S8. Accuracy of prediction for biome reconstructions

Assessment of accuracy for the two separate reconstructions together with details of which millennia have been used to train the model; n describes the number of ground truth points that were utilized in the accuracy assessment.

EXP	ORBIT	CO2 (PPM)	CH4 (PPB)	N2O (PPB)
**0**	0 ka	280	760	270
**1**	1 ka	279	627	262
**2**	2 ka	277	605	265
**3**	3 ka	275	572	268
**4**	4 ka	273	566	262
**5**	5 ka	268	559	260
**6**	6 ka	265	564	257
**7**	7 ka	261	607	261
**8**	8 ka	261	627	258
**9**	9 ka	265	666	259
**10**	10 ka	267	680	271
**11**	11 ka	264	670	267
**12**	12 ka	245	463	244
**13**	13 ka	238	655	262
**14**	14 ka	237	561	264
**15**	15 ka	224	472	241
**16**	16 ka	210	437	229
**17**	17 ka	194	377	237
**18**	18 ka	189	371	244
**19**	19 ka	188	375	219
**20**	20 ka	188	374	224
**21**	21 ka	186	365	245

Table S9. Boundary conditions for the HadCM3 simulation

Boundary conditions applied to the HadCM3 model, following Singarayer and Valdes (*71*). Orbital parameters are taken from Berger and Loutre (*119*), atmospheric CO_2 concentrations were taken from Vostok (*120*), and CH_4 and N_2O from EPICA (*121*).

REFERENCES AND NOTES

1. D. M. Sigman, M. P. Hain, G. H. Haug, The polar ocean and glacial cycles in atmospheric CO_2 concentration. *Nature* **466**, 47–55 (2010).

2. C. W. Snyder, Evolution of global temperature over the past two million years. *Nature* **538**, 226–228 (2016).

3. A. Schmittner, E. D. Galbraith, Glacial greenhouse-gas fluctuations controlled by ocean circulation changes. *Nature* **456**, 373–376 (2008).

4. E. D. Galbraith, L. C. Skinner, The biological pump during the Last Glacial Maximum. *Ann. Rev. Mar. Sci.* **12**, 559–586 (2020).

5. P. Ciais, A. Tagliabue, M. Cuntz, L. Bopp, M. Scholze, G. Hoffmann, A. Lourantou, S. P. Harrison, I. C. Prentice, D. I. Kelley, C. Koven, S. L. Piao, Large inert carbon pool in the terrestrial biosphere during the Last Glacial Maximum. *Nat. Geosci.* **5**, 74–79 (2011).

6. L. Menviel, J. Yu, F. Joos, A. Mouchet, K. J. Meissner, M. H. England, Poorly ventilated deep ocean at the Last Glacial Maximum inferred from carbon isotopes: A data-model comparison study. *Paleoceanography* **32**, 2–17 (2017).

7. C. D. Peterson, L. E. Lisiecki, J. V. Stern, Deglacial whole-ocean $\delta^{13}C$ change estimated from 480 benthic foraminiferal records. *Paleoceanography* **29**, 549–563 (2014).

8. C. D. Peterson, L. E. Lisiecki, Deglacial carbon cycle changes observed in a compilation of 127 benthic $\delta^{13}C$ time series (20–6 ka). *Clim. Past* **14**, 1229–1252 (2018).

9. S. A. Marcott, T. K. Bauska, C. Buizert, E. J. Steig, J. L. Rosen, K. M. Cuffey, T. J. Fudge, J. P. Severinghaus, J. Ahn, M. L. Kalk, J. R. McConnell, T. Sowers, K. C. Taylor, J. W. C. White, E. J. Brook, Centennial-scale changes in the global carbon cycle during the last deglaciation. *Nature* **514**, 616–619 (2014).

10. T. K. Bauska, D. Baggenstos, E. J. Brook, A. C. Mix, S. A. Marcott, V. V. Petrenko, H. Schaefer, J. P. Severinghaus, J. E. Lee, Carbon isotopes characterize rapid changes in

atmospheric carbon dioxide during the last deglaciation. *Proc. Natl. Acad. Sci. U.S.A.* **113**, 3465–3470 (2016).

11. J. M. Adams, H. Faure, L. Faure-Denard, J. M. McGlade, F. I. Woodward, Increases in terrestrial carbon storage from the Last Glacial Maximum to the present. *Nature* **348**, 711–714 (1990).

12. J. O. Kaplan, I. C. Prentice, W. Knorr, P. J. Valdes, Modeling the dynamics of terrestrial carbon storage since the Last Glacial Maximum. *Geophys. Res. Lett.* **29**, 10–13 (2002).

13. A. Lindgren, G. Hugelius, P. Kuhry, Extensive loss of past permafrost carbon but a net accumulation into present-day soils. *Nature* **560**, 219–222 (2018).

14. Z. Lu, P. A. Miller, Q. Zhang, D. Wårlind, L. Nieradzik, J. Sjolte, Q. Li, B. Smith, Vegetation pattern and terrestrial carbon variation in past warm and cold climates. *Geophys. Res. Lett.* **46**, 8133–8143 (2019).

15. I. C. Prentice, S. P. Harrison, P. J. Bartlein, Global vegetation and terrestrial carbon cycle changes after the last ice age. *New Phytol.* **189**, 988–998 (2011).

16. M. Winterfeld, G. Mollenhauer, W. Dummann, P. Köhler, L. Lembke-Jene, V. D. Meyer, J. Hefter, C. McIntyre, L. Wacker, U. Kokfelt, R. Tiedemann, Deglacial mobilization of pre-aged terrestrial carbon from degrading permafrost. *Nat. Commun.* **9**, 3666 (2018).

17. M. Turetsky, B. Abbott, M. Jones, K. M. Walter Anthony, D. Olefeldt, E. A. G. Schuur, G. Grosse, P. Kuhry, G. Hugelius, C. D. Koven, D. M. Lawrence, C. Gibson, A. Sannel, A. D. McGuire, Carbon release through abrupt permafrost thaw. *Nat. Geosci.* **13**, 138–143 (2020).

18. M. Fritz, J. E. Vonk, H. Lantuit, Collapsing Arctic coastlines. *Nat. Clim. Chang.* **7**, 6–7 (2017).

19. J. Martens, B. Wild, F. Muschitiello, M. O’Regan, M. Jakobsson, I. Semiletov, O. V. Dudarev, Ö. Gustafsson, Remobilization of dormant carbon from Siberian-Arctic permafrost during three past warming events. *Sci. Adv.* **6**, eabb6546 (2022).

20. A. Nogarotto, R. Noormets, T. Chauhan, G. Mollenhauer, J. Hefter, H. Grotheer, S. T. Belt, F. Colleoni, F. Muschitiello, L. Capotondi, C. Pellegrini, T. Tesi, Coastal permafrost was massively eroded during the Bølling-Allerød warm period. *Commun. Earth Environ.* **4**, 350 (2023).

21. B. Riddell-Young, J. E. Lee, E. J. Brook, J. Schmitt, H. Fischer, T. K. Bauska, J. A. Menking, R. Iseli, J. R. Clark, Abrupt changes in biomass burning during the last glacial period. *Nature* **637**, 91–96 (2025).

22. B. D. Stocker, Z. Yu, C. Massa, F. Joos, Holocene peatland and ice-core data constraints on the timing and magnitude of CO_2 emissions from past land use. *Proc. Natl. Acad. Sci. U.S.A.* **114**, 1492–1497 (2017).

23. A. Lindgren, "Northern permafrost region soil carbon dynamics since the Last Glacial Maximum: A terrestrial component in the glacial to interglacial carbon cycle," thesis, Department of Physical Geography, Stockholm (2020).

24. G. Hugelius, C. Tarnocai, G. Broll, J. G. Canadell, P. Kuhry, D. K. Swanson, The northern circumpolar soil carbon database: Spatially distributed datasets of soil coverage and soil carbon storage in the northern permafrost regions. *Earth Syst. Sci. Data* **5**, 3–13 (2013).

25. J. Ding, F. Li, G. Yang, L. Chen, B. Zhang, L. Liu, K. Fang, S. Qin, Y. Chen, Y. Peng, C. Ji, H. He, P. Smith, Y. Yang, The permafrost carbon inventory on the Tibetan Plateau: A new evaluation using deep sediment cores. *Glob. Chang. Biol.* **22**, 2688–2701 (2016).

26. D. M. Olson, E. Dinerstein, E. D. Wikramanayake, N. D. Burgess, G. V. N. Powell, E. C. Underwood, J. A. D'amico, I. Itoua, H. E. Strand, J. C. Morrison, C. J. Loucks, T. F. Allnutt, T. H. Ricketts, Y. Kura, J. F. Lamoreux, W. W. Wettengel, P. Hedao, K. R. Kassem, Terrestrial ecoregions of the world: A New map of life on Earth. *Bioscience* **51**, 933–938 (2001).

27. R. Zech, Y. Huang, M. Zech, R. Tarozo, W. Zech, High carbon sequestration in Siberian permafrost loess-paleosols during glacials. *Clim. Past* **7**, 501–509 (2011).

28. C. C. Treat, T. Kleinen, N. Broothaerts, A. S. Dalton, R. Dommaine, T. A. Douglas, J. Z. Drexler, S. A. Finkelstein, G. Grosse, G. Hope, J. Hutchings, M. C. Jones, P. Kuhry, T. Lacourse, O. Lähteenoja, J. Loisel, B. Notebaert, R. J. Payne, D. M. Peteet, A. B. K. Sannel, J. M. Stelling, J. Strauss, G. T. Swindles, J. Talbot, C. Tarnocai, G. Verstraeten, C. J. Williams, Z. Xia, Z. Yu, M. Väliranta, M. Hättestrand, H. Alexanderson, V. Brovkin, Widespread global peatland establishment and persistence over the last 130,000 y. *Proc. Natl. Acad. Sci. U.S.A.* **116**, 4822–4827 (2019).

29. Z. C. Yu, Northern peatland carbon stocks and dynamics: A review. *Biogeosciences* **9**, 4071–4085 (2012).

30. A. S. Dyke, J. T. Andrews, P. U. Clark, J. H. England, G. H. Miller, J. Shaw, J. J. Veillette, The Laurentide and Innuitian ice sheets during the Last Glacial Maximum. *Quat. Sci. Rev.* **21**, 9–31 (2002).

31. A. P. Stroeven, C. Hättestrand, J. Kleman, J. Heyman, D. Fabel, O. Fredin, B. W. Goodfellow, J. M. Harbor, J. D. Jansen, L. Olsen, M. W. Caffee, D. Fink, J. Lundqvist, G. C. Rosqvist, B. Strömberg, K. N. Jansson, Deglaciation of Fennoscandia. *Quat. Sci. Rev.* **147**, 91–121 (2016).

32. J. W. Harden, E. T. Sundquist, R. F. Stallard, R. K. Mark, Dynamics of soil carbon during deglaciation of the laurentide ice sheet. *Science* **258**, 1921–1924 (1992).

33. A. Montenegro, M. Eby, J. O. Kaplan, K. J. Meissner, A. J. Weaver, Carbon storage on exposed continental shelves during the glacial-interglacial transition. *Geophys. Res. Lett.* **33**, L08703 (2006).

34. V. Rachold, D. Y. Bolshiyanov, M. N. Grigoriev, H.-W. Hubberten, R. Junker, V. V. Kunitsky, F. Merker, P. Overduin, W. Schneider, Nearshore arctic subsea permafrost in transition. *Eos Trans. Am. Geophys. Union* **88**, 149–150 (2007).

35. S. S. Sayedi, B. W. Abbott, B. F. Thornton, J. M. Frederick, J. E. Vonk, P. Overduin, C. Schädel, E. A. G. Schuur, A. Bourbonnais, N. Demidov, A. Gavrilov, S. He, G. Hugelius, M. Jakobsson, M. C. Jones, D. Joung, G. Kraev, R. W. Macdonald, A. David McGuire, C. Mu, M. O'Regan, K. M. Schreiner, C. Stranne, E. Pizhankova, A. Vasiliev, S. Westermann, J. P.

Zarnetske, T. Zhang, M. Ghandehari, S. Baeumler, B. C. Brown, R. J. Frei, Subsea permafrost carbon stocks and climate change sensitivity estimated by expert assessment. *Environ. Res. Lett.* **15**, 124075 (2020).

36. J. E. Vonk, L. Sánchez-García, B. E. Van Dongen, V. Alling, D. Kosmach, A. Charkin, I. P. Semiletov, O. V. Dudarev, N. Shakhova, P. Roos, T. I. Eglinton, A. Andersson, Ö. Gustafsson, Activation of old carbon by erosion of coastal and subsea permafrost in Arctic Siberia. *Nature* **489**, 137–140 (2012).

37. C. Schädel, E. A. G. Schuur, R. Bracho, B. Elberling, C. Knoblauch, H. Lee, Y. Luo, G. R. Shaver, M. R. Turetsky, Circumpolar assessment of permafrost C quality and its vulnerability over time using long-term incubation data. *Glob. Chang. Biol.* **20**, 641–652 (2014).

38. G. Tanski, D. Wagner, C. Knoblauch, M. Fritz, T. Sachs, H. Lantuit, Rapid CO_2 release from eroding permafrost in seawater. *Geophys. Res. Lett.* **46**, 11244–11252 (2019).

39. R. F. Anderson, S. Ali, L. I. Bradtmiller, S. H. H. Nielsen, M. Q. Fleisher, B. E. Anderson, L. H. Burckle, Wind-driven upwelling in the southern ocean and the deglacial rise in atmospheric CO_2. *Science* **323**, 1443–1448 (2009).

40. A. Lindgren, G. Hugelius, P. Kuhry, T. R. Christensen, J. Vandenberghe, GIS-based maps and area estimates of northern hemisphere permafrost extent during the Last Glacial Maximum. *Permafr. Periglac. Process.* **27**, 6–16 (2015).

41. J. C. Walters, Ice-wedge casts and relict polygonal patterned ground in North-East Iowa, USA. *Permafr. Periglac. Process.* **5**, 269–282 (1994).

42. W. J. Wayne, Ice-wedge casts of Wisconsinan age in eastern Nebraska. *Permafr. Periglac. Process.* **2**, 211–223 (1991).

43. K. M. Walter, M. E. Edwards, G. Grosse, S. A. Zimov, F. S. Chapin III, Thermokarst lakes as a source. *Science* **318**, 633–636 (2007).

44. L. S. Brosius, K. M. Walter Anthony, C. C. Treat, M. C. Jones, M. Dyonisius, G. Grosse, Panarctic lakes exerted a small positive feedback on early Holocene warming due to deglacial release of methane. *Commun. Earth Environ.* **4**, 271 (2023).

45. M. N. Dyonisius, V. V. Petrenko, A. M. Smith, Q. Hua, B. Yang, J. Schmitt, J. Beck, B. Seth, M. Bock, B. Hmiel, I. Vimont, J. A. Menking, S. A. Shackleton, D. Baggenstos, T. K. Bauska, R. H. Rhodes, P. Sperlich, R. Beaudette, C. Harth, M. Kalk, E. J. Brook, H. Fischer, J. P. Severinghaus, R. F. Weiss, Old carbon reservoirs were not important in the deglacial methane budget. *Science* **367**, 907–910 (2020).

46. V. V. Petrenko, A. M. Smith, H. Schaefer, K. Riedel, E. Brook, D. Baggenstos, C. Harth, Q. Hua, C. Buizert, A. Schilt, X. Fain, L. Mitchell, T. Bauska, A. Orsi, R. F. Weiss, J. P. Severinghaus, Minimal geological methane emissions during the Younger Dryas–Preboreal abrupt warming event. *Nature* **548**, 443–446 (2017).

47. O. V. Masyagina, O. V. Menyailo, The impact of permafrost on carbon dioxide and methane fluxes in Siberia: A meta-analysis. *Environ. Res.* **182**, 109096 (2020).

48. F. Joos, R. Roth, J. S. Fuglestvedt, G. P. Peters, I. G. Enting, W. von Bloh, V. Brovkin, E. J. Burke, M. Eby, N. R. Edwards, T. Friedrich, T. L. Frölicher, P. R. Halloran, P. B. Holden, C. Jones, T. Kleinen, F. T. Mackenzie, K. Matsumoto, M. Meinshausen, G.-K. Plattner, A. Reisinger, J. Segschneider, G. Shaffer, M. Steinacher, K. Strassmann, K. Tanaka, A. Timmermann, A. J. Weaver, Carbon dioxide and climate impulse response functions for the computation of greenhouse gas metrics: A multi-model analysis. *Atmos. Chem. Phys.* **13**, 2793–2825 (2013).

49. D. Archer, V. Brovkin, The millennial atmospheric lifetime of anthropogenic CO_2. *Clim. Change* **90**, 283–297 (2008).

50. Z. Yu, F. Joos, T. K. Bauska, B. D. Stocker, H. Fischer, J. Loisel, V. Brovkin, G. Hugelius, C. Nehrbass-Ahles, T. Kleinen, J. Schmitt, No support for carbon storage of >1,000 GtC in northern peatlands. *Nat. Geosci.* **14**, 465–467 (2021).

51. J. Elsig, J. Schmitt, D. Leuenberger, R. Schneider, M. Eyer, M. Leuenberger, F. Joos, H. Fischer, T. F. Stocker, Stable isotope constraints on Holocene carbon cycle changes from an Antarctic ice core. *Nature* **461**, 507–510 (2009).

52. G. Grassi, C. Schwingshackl, T. Gasser, R. A. Houghton, S. Sitch, J. G. Canadell, A. Cescatti, P. Ciais, S. Federici, P. Friedlingstein, W. A. Kurz, M. J. Sanz Sanchez, R. Abad Viñas, R. Alkama, S. Bultan, G. Ceccherini, S. Falk, E. Kato, D. Kennedy, J. Knauer, A. Korosuo, J. Melo, M. J. McGrath, J. E. M. S. Nabel, B. Poulter, A. A. Romanovskaya, S. Rossi, H. Tian, A. P. Walker, W. Yuan, X. Yue, J. Pongratz, Harmonising the land-use flux estimates of global models and national inventories for 2000–2020. *Earth Syst. Sci. Data* **15**, 1093–1114 (2023).

53. J. Gottschalk, L. C. Skinner, S. L. Jaccard, L. Menviel, C. Nehrbass-Ahles, C. Waelbroeck, Southern Ocean link between changes in atmospheric CO_2 levels and northern-hemisphere climate anomalies during the last two glacial periods. *Quat. Sci. Rev.* **230**, 106067 (2020).

54. T. K. Bauska, E. J. Brook, S. A. Marcott, D. Baggenstos, S. Shackleton, J. P. Severinghaus, V. V. Petrenko, Controls on millennial-scale atmospheric CO_2 variability during the last glacial period. *Geophys. Res. Lett.* **45**, 7731–7740 (2018).

55. M. Claussen, A. Dallmeyer, J. Bader, *Theory and Modeling of the African Humid Period and the Green Sahara* (Oxford Univ. Press USA, 2017); http://climatescience.oxfordre.com/view/10.1093/acrefore/9780190228620.001.0001/acrefore-9780190228620-e-532, vol. 1.

56. A. W. Jacobel, J. F. McManus, R. F. Anderson, G. Winckler, Large deglacial shifts of the Pacific Intertropical Convergence Zone. *Nat. Commun.* **7**, 10449 (2016).

57. R. Dommain, J. Couwenberg, P. H. Glaser, H. Joosten, I. N. N. Suryadiputra, Carbon storage and release in Indonesian peatlands since the last deglaciation. *Quat. Sci. Rev.* **97**, 1–32 (2014).

58. J. F. Abrams, S. Hohn, T. Rixen, A. Merico, Sundaland peat carbon dynamics and its contribution to the Holocene atmospheric $_{CO2}$ concentration. *Global Biogeochem. Cycles* **32**, 704–719 (2018).

59. J. Schmitt, R. Schneider, J. Elsig, D. Leuenberger, A. Lourantou, J. Chappellaz, P. Kohler, F. Joos, T. F. Stocker, M. Leuenberger, H. Fischer, Carbon isotope constraints on the deglacial CO_2 rise from ice cores. *Science* **336**, 711–714 (2012).

60. F. He, “Simulating transient climate evolution of the last deglaciation with CCSM3,” thesis, University of Wisconsin-Madison (2011).

61. A. Lindgren, P. Kuhry, M. Holloway, Z. Lu, G. Hugelius, Soil carbon modelling from the Last Glacial Maximum (LGM) to present for the Northern Hemisphere—Input data. Bolin Data Center (2025); https://doi.org/10.17043/lindgren-2025-carbon-model-input-1.

62. Z. Liu, B. L. Otto-Bliesner, F. He, E. C. Brady, R. Tomas, P. U. Clark, A. E. Carlson, J. Lynch-Stieglitz, W. Curry, E. Brook, D. Erickson, R. Jacob, J. Kutzbach, J. Cheng, Transient simulation of last deglaciation with a new mechanism for bolling-allerod warming. *Science* **325**, 310–314 (2009).

63. A. L. Berger, Long-term variations of daily insolation and quaternary climatic changes. *J. Atmos. Sci.* **35**, 2362–2367 (1978).

64. F. Joos, R. Spahni, Rates of change in natural and anthropogenic radiative forcing over the past 20,000 years. *Proc. Natl. Acad. Sci. U.S.A.* **105**, 1425–1430 (2008).

65. J. F. McManus, R. Francois, J. M. Gherardl, L. Kelgwin, S. Drown-Leger, Collapse and rapid resumption of Atlantic meridional circulation linked to deglacial climate changes. *Nature* **428**, 834–837 (2004).

66. W. R. Peltier, Global glacial isostacy and the surface of the ice-age Eart: The ICE-5G (VM2) model and GRACE. *Annu. Rev. Earth Planet. Sci.* **32**, 111–149 (2004).

67. F. He, J. D. Shakun, P. U. Clark, A. E. Carlson, Z. Liu, B. L. Otto-bliesner, J. E. Kutzbach, Northern Hemisphere forcing of Southern Hemisphere climate during the last deglaciation. *Nature* **494**, 81–85 (2013).

68. Z. Liu, J. Zhu, Y. Rosenthal, X. Zhang, B. L. Otto-bliesner, A. Timmermann, R. S. Smith, The Holocene temperature conundrum. *Proc. Natl. Acad. Sci. U.S.A.* **111**, E3501–E3505 (2014).

69. B. L. Otto-bliesner, J. M. Russell, P. U. Clark, Z. Liu, J. T. Overpeck, B. Konecky, S. E. Nicholson, F. He, Z. Lu, Coherent changes of southeastern equatorial and northern African rainfall during the last deglaciation. *Science* **346**, 1223–1228 (2014).

70. X. Wen, Z. Liu, S. Wang, J. Cheng, J. Zhu, Correlation and anti-correlation of the East Asian summer and winter monsoons during the last 21,000 years. *Nat. Commun.* **7**, 11999 (2016).

71. J. S. Singarayer, P. J. Valdes, High-latitude climate sensitivity to ice-sheet forcing over the last 120 kyr. *Quat. Sci. Rev.* **29**, 43–55 (2010).

72. T. Hengl, M. G. Walsh, J. Sanderman, I. Wheeler, S. P. Harrison, I. C. Prentice, Global mapping of potential natural vegetation: An assessment of machine learning algorithms for estimating land potential. *PeerJ* **6**, e5457 (2018).

73. F. Pedregosa, G. Varoquaux, A. Gramfort, V. Michel, B. Thirion, O. Grisel, M. Blondel, P. Prettenhofer, R. Weiss, V. Dubourg, J. Vanderplas, A. Passos, D. Cournapeau, M. Brucher, M. Perrot, É. Deuchesnay, Scikit-learn: Machine Learning in Python. *J. Mach. Learn. Res.* **12**, 2825–2830 (2011).

74. A. Lindgren, Z. Lu, Q. Zhang, G. Hugelius, Reconstructing past global vegetation with random forest machine learning, sacrificing the dynamic response for robust results. *J. Adv. Model. Earth Syst.* **13**, e2020MS002200 (2021).

75. H. Binney, M. Edwards, M. Macias-Fauria, A. Lozhkin, P. Anderson, J. O. Kaplan, A. Andreev, E. Bezrukova, T. Blyakharchuk, V. Jankovska, I. Khazina, S. Krivonogov, K. Kremenetski, J. Nield, E. Novenko, N. Ryabogina, N. Solovieva, K. Willis, V. Zernitskaya, Vegetation of Eurasia from the last glacial maximum to present: Key biogeographic patterns. *Quat. Sci. Rev.* **157**, 80–97 (2017).

76. A. S. Dyke, Late quaternary vegetation history of northern North America based on pollen, macrofossil, and faunal remains. *Géog. Phys. Quatern.* **59**, 211–262 (2005).

77. S. P. Harrison, Biome 6000 DB classified plotfile version 1 (University of Reading, 2017); https://doi.org/10.17864/1947.99.

78. J. Ni, G. Yu, S. P. Harrison, I. C. Prentice, Palaeovegetation in China during the late Quaternary: Biome reconstructions based on a global scheme of plant functional types. *Palaeogeogr. Palaeoclimatol. Palaeoecol.* **289**, 44–61 (2010).

79. A. Lindgren, Soil carbon modelling from the Last Glacial Maximum (LGM) to present for the Northern Hemisphere. Bolin Center Data Repository 1.0.0 (2025); https://doi.org/10.57669/lindgren-2025-carbon-model-1.0.0.

80. N. Ray, J. M. Adams, A GIS-based vegetation map of the world at the Last Glacial Maximum (25,000-15,000 BP). *Internet Archaeol.* **11**, 0–15 (2001).

81. A. Lindgren, P. Kuhry, M. Holloway, Z. Lu, G. Hugelius, Soil carbon modelling from the Last Glacial Maximum (LGM) to present for the Northern Hemisphere—Output data. Bolin Center Database (2025); https://doi.org/10.17043/lindgren-2025-carbon-model-output-1.

82. N. H. Batjes, Harmonized soil property values for broad-scale modelling (WISE30sec) with estimates of global soil carbon stocks. *Geoderma* **269**, 61–68 (2016).

83. K. L. Verdin, "ISLSCP II HYDRO1k Elevation-derived Products, in *ISLSCP Initiative II Collection. Data Set*, F. G. Hall, G. Collatz, B. Meeson, S. Los, E. Brown de Colstoun, D. Landis, Eds. (2011). [http://daac.ornl.gov/] from Oak Ridge National Laboratory Distributed Active Archive Center, Oak Ridge, Tennessee, USA. 10.3334/ORNLDAAC/1007.

84. M. Fuchs, P. Kuhry, G. Hugelius, Low below-ground organic carbon storage in a subarctic Alpine permafrost environment. *Cryosphere* **9**, 427–438 (2015).

85. S. E. Chadburn, E. J. Burke, P. M. Cox, P. Friedlingstein, G. Hugelius, S. Westermann, An observation-based constraint on permafrost loss as a function of global warming. *Nat. Clim. Chang.* **7**, 340–344 (2017).

86. J. Brown, O. Ferrains, J. Heginbottom, E. Melnikov, Circum-Arctic Map of Permafrost and Ground-Ice Conditions, Version 2. Boulder, Colorado, USA. Nasa National Snow and Ice Data Center Distributed Active Archive Center (2002). https://nsidc.org/data/ggd318/versions/2.

87. V. V. Baulin, Ye. B. Belopukhova, N. S. Danilova, "Holocene permafrost in the USSR" in *Late Quaternary Environments of the Soviet Union*, A. A. Velichko, Ed. (University of Minnesota Press, 1984), pp. 87–91.

88. S. C. Zoltai, Permafrost distribution in peatlands of west-central Canada during the Holocene warm period 6000 years BP. *Geogr. Phys. Quatern.* **49**, 37–43 (1995).

89. B. Elberling, A. Michelsen, C. Schädel, E. A. G. Schuur, H. H. Christiansen, L. Berg, M. P. Tamstorf, C. Sigsgaard, Long-term CO_2 production following permafrost thaw. *Nat. Clim. Chang.* **3**, 890–894 (2013).

90. J. Chlachula, The Siberian loess record and its significance for reconstruction of Pleistocene climate change in north-central Asia. *Quat. Sci. Rev.* **22**, 1879–1906 (2003).

91. P. Haesaerts, I. Borziak, L. Koulakovska, V. Chirica, F. Damblon, J. van der Plicht, The east Carpathian loess record: A reference for the middle and late pleniglacial stratigraphy in central Europe. *Quaternaire* **14**, 163–188 (2003).

92. D. R. Muhs, T. A. Ager, G. Skipp, J. Beann, J. Budahn, P. Mcgeehin, D. R. Muhs, T. A. Ager, G. Skipp, J. Beann, J. Budahri, J. P. Mcgeehin, Paleoclimatic significance of chemical weathering in loess-derived paleosols of subarctic central Alaska. *Arct. Antarct. Alp. Res.* **40**, 396–411 (2008).

93. D.-D. Rousseau, J.-J. Puisségur, A 350,000-year climatic record from the loess sequence of Achenheim, Alsace, France. *Boreas* **18**, 203–216 (1990).

94. J. Loisel, S. van Bellen, L. Pelletier, J. Talbot, G. Hugelius, D. Karran, Z. Yu, J. Nichols, J. Holmquist, Insights and issues with estimating northern peatland carbon stocks and fluxes since the Last Glacial Maximum. *Earth Sci. Rev.* **165**, 59–80 (2017).

95. G. Hugelius, J. Loisel, S. Chadburn, R. B. Jackson, M. Jones, G. MacDonald, M. Marushchak, D. Olefeldt, M. Packalen, M. B. Siewert, Large stocks of peatland carbon and nitrogen are vulnerable to permafrost thaw. *Proc. Natl. Acad. Sci. U.S.A.* **117**, 20438–20446 (2020).

96. E. Gorham, Northern peatlands: Role in the carbon cycle and probable responses to climatic warming. *Ecol. Appl.* **1**, 182–195 (1991).

97. P. J. Reimer, E. Bard, A. Bayliss, J. W. Beck, P. G. Blackwell, C. B. Ramsey, C. E. Buck, H. Cheng, R. L. Edwards, M. Friedrich, P. M. Grootes, T. P. Guilderson, H. Haflidason, I. Hajdas, C. Hatté, T. J. Heaton, D. L. Hoffmann, A. G. Hogg, K. A. Hughen, K. F. Kaiser, B. Kromer, S. W. Manning, M. Niu, R. W. Reimer, D. A. Richards, E. M. Scott, J. R. Southon, R. A. Staff, C. S. M. Turney, J. van der Plicht, IntCal13 and Marine13 radiocarbon age calibraion cuvres 0–50,000 years cal BP. *Radiocarbon* **55**, 1869–1887 (2013).

98. A. V. Kislov, A. Panin, P. Toropov, Current status and palaeostages of the Caspian Sea as a potential evaluation tool for climate model simulations. *Quat. Int.* **345**, 48–55 (2014).

99. B. W. Goodfellow, Relict non-glacial surfaces in formerly glaciated landscapes. *Earth Sci. Rev.* **80**, 47–73 (2007).

100. P. R. Bierman, L. B. Corbett, J. A. Graly, T. A. Neumann, A. Lini, B. T. Crosby, D. H. Rood, Preservation of a preglacial landscape under the center of the Greenland ice sheet. *Science* **344**, 402–405 (2014).

101. G. Hugelius, J. Strauss, S. Zubrzycki, J. W. Harden, E. A. G. Schuur, C. L. Ping, L. Schirrmeister, G. Grosse, G. J. Michaelson, C. D. Koven, J. A. O'Donnell, B. Elberling, U. Mishra, P. Camill, Z. Yu, J. Palmtag, P. Kuhry, Estimated stocks of circumpolar permafrost carbon with quantified uncertainty ranges and identified data gaps. *Biogeosciences* **11**, 6573–6593 (2014).

102. J. Kleman, C. Hattestrand, Frozen-bed Fennoscandian and Laurentide ice sheets during the Last Glacial Maximum. *Nature* **402**, 63–66 (1999).

103. J. L. Wadham, J. R. Hawkings, L. Tarasov, L. J. Gregoire, R. G. M. Spencer, M. Gutjahr, A. Ridgwell, K. E. Kohfeld, Ice sheets matter for the global carbon cycle. *Nat. Commun.* **10**, 3567 (2019).

104. S. Höfle, J. Rethemeyer, C. W. Mueller, S. John, Organic matter composition and stabilization in a polygonal tundra soil of the Lena Delta. *Biogeosciences* **10**, 3145–3158 (2013).

105. J. E. Vonk, I. P. Semiletov, O. V. Dudarev, T. I. Eglinton, A. Anderson, N. Shakhova, A. Charkin, B. Heim, Ö. Gustafsson, Preferential burial of permafrost-derived organic carbon in Siberian-Arctic shelf waters. *J. Geophys. Res. Oceans* **119**, 8410–8421 (2014).

106. L. Bröder, T. Tesi, A. Andersson, I. Semiletov, Ö. Gustafsson, Bounding cross-shelf transport time and degradation in Siberian-Arctic land-ocean carbon transfer. *Nat. Commun.* **9**, 806 (2018).

107. H. Grotheer, V. Meyer, T. Riedel, G. Pfalz, L. Mathieu, J. Hefter, T. Gentz, H. Lantuit, G. Mollenhauer, M. Fritz, Burial and origin of permafrost-derived carbon in the nearshore zone of the southern Canadian Beaufort Sea. *Geophys. Res. Lett.* **47**, e2019GL085897 (2020).

108. C. Knoblauch, C. Beer, A. Sosnin, D. Wagner, E. Pfeiffer, Predicting long-term carbon mineralization and trace gas production from thawing permafrost of Northeast Siberia. *Glob. Chang. Biol.* **19**, 1160–1172 (2013).

109. E. A. G. Schuur, A. D. McGuire, C. Schädel, G. Grosse, J. W. Harden, D. J. Hayes, G. Hugelius, C. D. Koven, P. Kuhry, D. M. Lawrence, Climate change and the permafrost carbon feedback. *Nature* **520**, 171–179 (2015).

110. B. W. Abbott, J. B. Jones, Permafrost collapse alters soil carbon stocks, respiration, CH_4, and N_2O in upland tundra. *Glob. Chang. Biol.* **21**, 4570–4587 (2015).

111. D. M. Nielsen, M. Dobrynin, J. Baehr, S. Razumov, M. Grigoriev, Coastal erosion variability at the southern Laptev Sea linked to winter sea ice and the Arctic Oscillation. *Geophys. Res. Lett.* **47**, e2019GL086876 (2020).

112. E. S. Karlsson, V. Brüchert, T. Tesi, A. Charkin, O. Dudarev, I. Semiletov, Ö. Gustafsson, Contrasting regimes for organic matter degradation in the East Siberian Sea and the Laptev Sea assessed through microbial incubations and molecular markers. *Mar. Chem.* **170**, 11–22 (2015).

113. I. Semiletov, I. Pipko, Ö. Gustafsson, L. G. Anderson, V. Sergienko, S. Pugach, O. Dudarev, A. Charkin, A. Gukov, L. Bröder, Acidification of East Siberian Arctic Shelf waters through addition of freshwater and terrestrial carbon. *Nat. Geosci.* **9**, 361–365 (2016).

114. I. I. Pipko, S. P. Pugach, I. P. Semiletov, L. G. Anderson, N. E. Shakhova, Ö. Gustafsson, I. A. Repina, E. A. Spivak, A. N. Charkin, A. N. Salyuk, The spatial and interannual dynamics of the surface water carbonate system and air–sea CO_2 fluxes in the outer shelf and slope of the Eurasian Arctic Ocean. *Ocean Sci.* **13**, 997–1016 (2017).

115. J. T. Mathis, R. S. Pickart, R. H. Byrne, C. L. McNeil, G. W. K. Moore, L. W. Juranek, X. Liu, J. Ma, R. A. Easley, M. M. Elliot, Storm-induced upwelling of high $p\mathrm{CO}_2$ waters onto the continental shelf of the western Arctic Ocean and implications for carbonate mineral saturation states. *Geophys. Res. Lett.* **39**, L07606 (2012).

116. T. S. Bianchi, The role of terrestrially derived organic carbon in the coastal ocean: A changing paradigm and the priming effect. *Proc. Natl. Acad. Sci. U.S.A.* **108**, 19473–19481 (2011).

117. E. Monnin, EPICA Dome C high resolution carbon dioxide concentrations. PANGAEA [Preprint] (2006); https://doi.org/10.1594/PANGAEA.472488.

118. J. Beck, M. Bock, J. Schmitt, B. Seth, T. Blunier, H. Fischer, C. J. Beck, Bipolar carbon and hydrogen isotope constraints on the Holocene methane budget. *Biogeosciences* **15**, 7155–7175 (2018).

119. A. Berger, M. F. Loutre, Insolation values for the climate of the last 10 million years. *Quat. Sci. Rev.* **10**, 297–317 (1991).

120. J. R. Petit, J. Jouzel, D. Raynaud, N. I. Barkov, J.-M. Barnola, I. Basile, M. Bender, J. Chappellaz, M. Davis, G. Delaygue, M. Delmotte, V. M. Kotlyakov, M. Legrand, V. Y. Lipenkov, C. Lorius, L. PÉpin, C. Ritz, E. Saltzman, M. Stievenard, Climate and atmospheric history of the past 420,000 years from the Vostok ice core, Antarctica. *Nature* **399**, 429–436 (1999).

121. R. Spahni, J. Chappellaz, T. F. Stocker, L. Loulergue, G. Hausammann, K. Kawamura, J. Flückiger, J. Schwander, D. Raynaud, V. Masson-Delmotte, J. Jouzel, Atmospheric methane and nitrous oxide of the late Pleistocene from Antarctic ice cores. *Science* **310**, 1317–1322 (2005).
